# Intrathecal Immunoglobulin for treatment of adult patients with tetanus: A randomized controlled 2x2 factorial trial

**DOI:** 10.12688/wellcomeopenres.14587.2

**Published:** 2018-11-05

**Authors:** Huỳnh Thị Loan, Lam Minh Yen, Evelyne Kestelyn:, Nguyen Van Hao, Tran Tan Thanh, Nguyen Thi Phuong Dung, Hugo C. Turner, Ronald B. Geskus, Marcel Wolbers, Le Van Tan, H. Rogier Van Doorn, Nicholas P. Day, Duncan Wyncoll, Tran Tinh Hien, Guy E. Thwaites, Nguyen Van Vinh Chau, C. Louise Thwaites

**Affiliations:** 1 Hospital for Tropical Diseases, Ho Chi Minh City, Vietnam; 2 Oxford University Clinical Research Unit, Hospital for Tropical Diseases, Ho Chi Minh City, Vietnam; 3 Centre for Tropical Medicine and Global Health, University of Oxford, Oxford, OX3 7FZ, UK; 4 Medicine and Pharmacy, Hong Bang International University, Ho Chi Minh City, Vietnam; 5 Mahidol Oxford Research Unit, Bangkok, 10400, Thailand; 6 Guys and St Thomas’ Hospitals NHS Foundation Trust, London, SE1 7EH, UK

**Keywords:** Tetanus, management, treatment, intrathecal, antitoxin, human tetanus immunoglobulin

## Abstract

Despite long-standing availability of an effective vaccine, tetanus remains a significant problem in many countries. Outcome depends on access to mechanical ventilation and intensive care facilities and in settings where these are limited, mortality remains high. Administration of tetanus antitoxin by the intramuscular route is recommended treatment for tetanus, but as the tetanus toxin acts within the central nervous system, it has been suggested that intrathecal administration of antitoxin may be beneficial. Previous studies have indicated benefit, but with the exception of one small trial no blinded studies have been performed.

The objective of this study is to establish whether the addition of intrathecal tetanus antitoxin reduces the need for mechanical ventilation in patients with tetanus. Secondary objectives: to determine whether the addition of intrathecal tetanus antitoxin reduces autonomic nervous system dysfunction and length of hospital/ intensive care unit stay; whether the addition of intrathecal tetanus antitoxin in the treatment of tetanus is safe and cost-effective; to provide data to inform recommendation of human rather than equine antitoxin.

This study will enroll adult patients (≥16 years old) with tetanus admitted to the Hospital for Tropical Diseases, Ho Chi Minh City. The study is a 2x2 factorial blinded randomized controlled trial. Eligible patients will be randomized in a 1:1:1:1 manner to the four treatment arms (intrathecal treatment and human intramuscular treatment, intrathecal treatment and equine intramuscular treatment, sham procedure and human intramuscular treatment, sham procedure and equine intramuscular treatment). Primary outcome measure will be requirement for mechanical ventilation. Secondary outcome measures: duration of hospital/ intensive care unit stay, duration of mechanical ventilation, in-hospital and 240-day mortality and disability, new antibiotic prescription, incidence of ventilator associated pneumonia and autonomic nervous system dysfunction, total dose of benzodiazepines and pipecuronium, and incidence of adverse events.

**Trial registration:** ClinicalTrials.gov NCT02999815

**Registration date: **21 December 2016

**Abbreviations:** CSF, cerebrospinal fluid; ICU, intensive care unit; IU, international units; AE, adverse event; SAE, serious adverse event; UAE, unexpected adverse event; ADL, activities of daily living; QALY, quality adjusted life year; OUCRU, Oxford University Clinical Research Unit

## Introduction

Tetanus is a vaccine-preventable disease that continues to occur despite several decades of sustained global health programs. Recently outbreaks of tetanus have been reported after natural disasters such as tsunamis and earthquakes ^1^. The true global burden of disease is unknown as reliable figures are only collected for cases of neonatal tetanus, but in 2015 the disease caused an estimated 48,199 to 80,042 deaths ^2^. Most of these deaths were in South and Southeast Asia and Sub-Saharan Africa, thus it is likely that a significant proportion of the population in these regions remains vulnerable to tetanus.

Severe tetanus, with frequent muscle spasm requires intensive care unit (ICU) treatment as patients require paralysis and mechanical ventilation to overcome muscle spasms. With good ICU management the mortality rate from tetanus can be reduced significantly, however these facilities are often unavailable in settings where most tetanus occurs ^3^. Even if they are available, patients require expensive and prolonged ICU stays with long periods of mechanical ventilation and its attending complications ^4, 5^. A recent review of outcomes in Africa highlighted the continuing high mortality rates and attributed this to the inability to access ICU facilities even if they were present ^6^.

Reducing the requirement for mechanical ventilation or shortening ICU stay should improve outcome of tetanus in all settings.

Standard tetanus treatment regimens include the administration of intramuscular tetanus antitoxin ^7^. Administration of tetanus antitoxin using the intrathecal route offers a potential benefit in treating severe tetanus as tetanus toxin acts within the central nervous system (CNS). Case series and small randomized trials have reported large ( some >50%) improvements in mortality and hospital stay in both adults and neonates treated with intrathecal antitoxin ^8– 19^. However, most reports are of poor quality with large methodological differences and possible biases. Only one blinded trial has ever been performed ^12^ recruiting a total of only 36 patients. A meta-analysis of 942 patients from randomized trials concluded the combined relative risk for mortality of intrathecal versus intramuscular antitoxin was 0.71 (95% CI 0.62–0.81) ^20^. However this meta-analysis contains some serious methodological errors and two recent reviews have concluded that there is still insufficient evidence regarding its use ^21, 22^.

Current treatment for tetanus in Vietnam consists of intramuscular equine antitoxin. One randomized controlled trial has compared equine antitoxin in the treatment of tetanus with human antitoxin (human tetanus immunoglobulin) in a total of 130 neonates. There was no difference in complications and no side effects attributable to antitoxin were apparent in either group ^23^. Currently in Vietnam, human antitoxin is more expensive and is only just becoming available, although it is the product recommended by the World Health Organization due to a theoretical improved side-effect profile (with a reduced risk of serum sickness and anaphylactic reactions) ^7, 24^. Better understanding of the side-effect profile and use of human versus equine preparations would be useful in Vietnam and many low and middle income countries when choosing which preparation to use. 

The primary aim of this study is to assess the benefit of additional intrathecal tetanus antitoxin to intramuscular regimens. However, due to the factorial design of this study, we are also able to compare the side effect profile of equine and human antitoxin.

## Protocol

**Figure 1.**
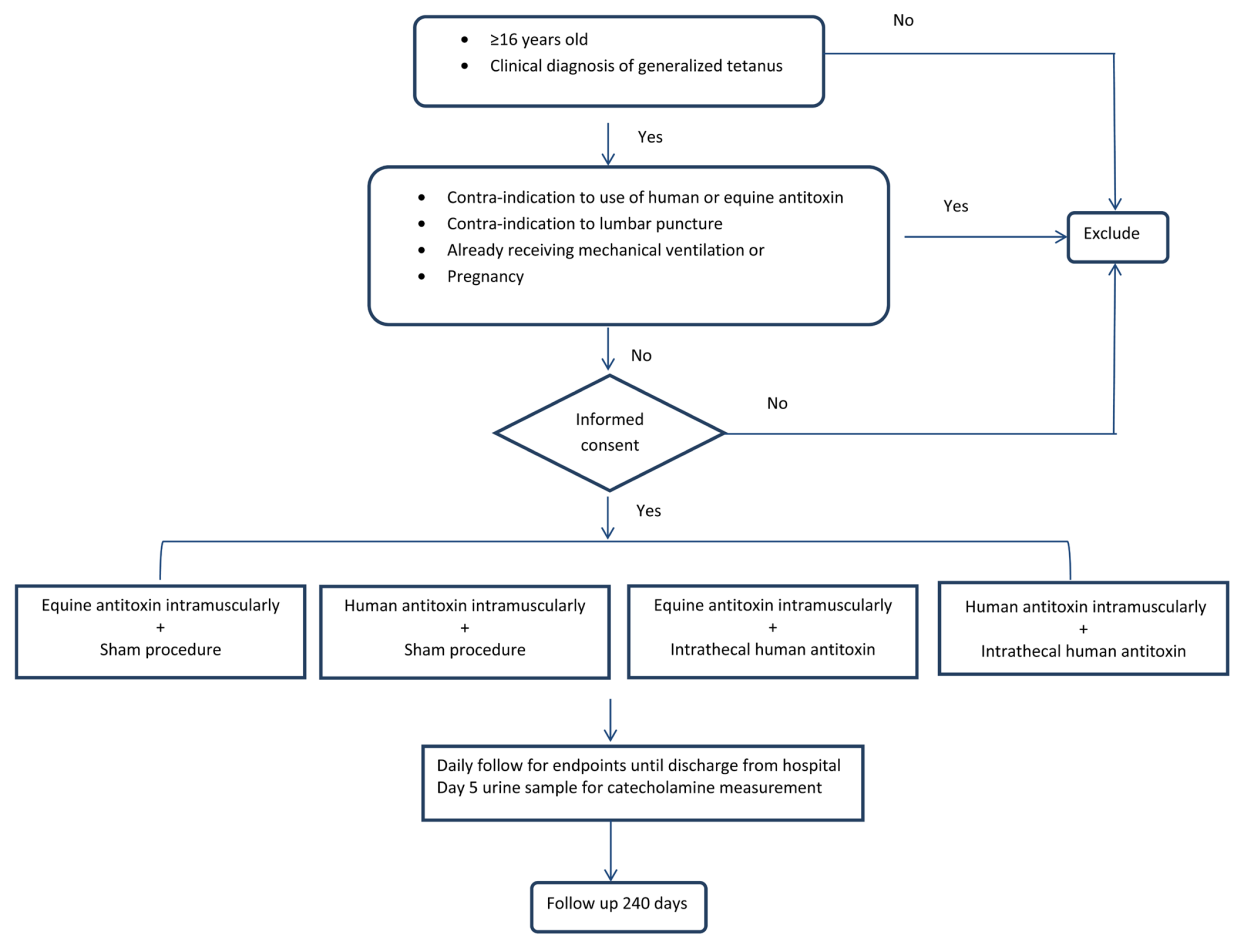
Study flowchart.

This protocol has been written according to the SPIRIT guidelines, see Supplementary File 1 and Supplementary File 2. Figure 1 shows the study flowchart.

### 1 Objectives

***1.1 Primary objective.*** To establish whether the addition of intrathecal tetanus antitoxin reduces the need for mechanical ventilation in patients with tetanus.

***1.2 Secondary objectives***

• To determine whether the addition or intrathecal tetanus antitoxin reduces autonomic nervous system dysfunction and length of hospital/ICU stay.
• To establish whether the addition of intrathecal tetanus antitoxin in the form of Tetagam-P is safe and cost-effective.
• To provide data to inform the recommendation of human rather than equine antitoxin

### 2 Study design

We will conduct a randomised partially-blinded controlled 2#215;2 factorial trial. First, adults admitted to the ICU at the Hospital for Tropical Diseases will be randomized to receive either human (3000 IU) or equine (21,000 units) intramuscular antitoxin. Second, participants will be randomized to receive either standard treatment with intramuscular antitoxin alone or with the addition of 500 IU intrathecal human antitoxin. Patients with contra-indications to lumbar puncture or antitoxin treatment will be excluded. Patients who have already received a treatment dose of intramuscular antitoxin will have the intramuscular injection omitted.

All patients will receive other standard tetanus treatment as deemed necessary by the attending physicians. Spasms will be treated with benzodiazepines as first-line therapy. Patients with spasms not controlled with benzodiazepines will receive tracheostomy, paralysis, magnesium sulphate and mechanical ventilation. Heart rate, blood pressure, respiratory rate, temperature, oxygen saturation and daily drug use will be recorded throughout the ICU stay. Patients will be followed following discharge from hospital until 240 days for disability/ death.

***2.1 Primary end point***

• Requirement for mechanical ventilation during ICU stay

Criteria for mechanical ventilation are SpO _2_ <90%; or PaO _2_/F _i_0 _2_ <250; or excessive spasms necessitating muscle paralysis. 

These criteria are intended as a guide and the final decision to ventilate a patient rests with the individual doctor responsible for the patient.

***2.2 Secondary End Points***

• Duration of ICU stay
• Duration of hospital stay
• Duration of mechanical ventilation
• In hospital and 240-day mortality and disability
• New antibiotic prescription during ICU stay (excluding antibiotics for tetanus or initial entry site infection)
• Incidence of Ventilator Associated Pneumonia
• Incidence of the clinical syndrome of autonomic nervous system dysfunction
• Total dose of benzodiazepines and pipecuronium during hospital stay
• Incidence of adverse events

### 3 Study procedures

***3.1 Entry criteria***

• All adult patients (≥16 years old) with a clinical diagnosis of generalized tetanus [as opposed to localized tetanus] admitted to the ICU at the Hospital for Tropical Diseases, Ho Chi Minh City, Vietnam.

***3.2 Exclusion criteria***

• Contra-indication to use of human or equine antitoxin
• Contra-indication to lumbar puncture
• Already receiving mechanical ventilation or expected to require this before intrathecal injection can be given
• Pregnancy
• Informed consent not obtained

***3.3 Patient identification.*** All patients with generalized tetanus will be identified by doctors working in the Hospital for Tropical Diseases ICU.

***3.4 Informed consent.*** Informed consent will be taken by the attending doctors, all of whom will receive specific training in the study and Good Clinical Practice and will be authorised to take consent by the trial principal investigator (see Supplementary File 3). These doctors will also assess whether or not the patient has mental capacity to provide informed consent. If the doctor judges that the patient does not have this capacity, they will obtain informed consent from the patient’s representative (usually a relative). It will be made completely and unambiguously clear that the patient (or their representative) is free to refuse to participate in all or any aspect of the trial, at any time and for any reason, without incurring any penalty or affecting their treatment.

The informed consent form will be presented to the participants or representatives detailing no less than: the exact nature of the study; the implications and constraints of the protocol; the known side effects, risks involved and alternatives to taking part. Those who refuse consent will be treated as per the best available standard of care and will not have any study related procedures performed. 

The patient or their representative must personally sign and date two of the latest approved versions of the informed consent form. The study staff will also sign and date the two copies. The patient/representative will receive one copy.

If the patient/representative is illiterate, a witness who is not a member of the study staff will be present during the informed consent discussion. The informed consent form will be read to the patient/representative in the presence of the witness. If the patient/representative agrees to participate, the form will be signed and dated by the witness. If the patient is a minor (defined as < 18 years of age) assent will need to be obtained in addition to parental or guardian consent.

If consent is provided by a representative and the patient regains the capacity to consider participation during the study period, the patient should be consulted and informed consent to continue the study obtained. If the patient refuses to give informed consent to participate they will be withdrawn from the study without compromise to their clinical care.

***3.5 Screening and eligibility assessment.*** Potential participants will be screened by the attending physicians. Screening will include clinical diagnosis and inspection of clinical notes. Results of any tests performed for clinical care during this illness episode may be used for the purposes of screening. A screening log will be kept on the ward, with a record of all patients screened and how they met/did not meet the study entry and exclusion criteria. No identifying details such as name will be recorded in this log. Patients who do not meet the study criteria will be informed as such and treated as per best available clinical care.

***3.6 Randomisation and treatment allocation.*** Randomisation will be 1:1:1:1 to the four treatment arms (intrathecal treatment and human intramuscular treatment, intrathecal treatment and equine intramuscular treatment, sham procedure and human intramuscular treatment, sham procedure and equine intramuscular treatment). Randomization will be based on a computer-based randomization list using block randomization with variable blocks lengths of 8 and 12 without stratification.

Only the study pharmacist who is not otherwise involved in the trial will have access to the randomization list and will use it to prepare treatment packages with sequential numbering. All treatment packs will be identical externally. Each patient will receive the next sequential package, which will be prepared in advance and available on the ward and stored appropriately. Each treatment pack will contain the appropriate study treatment. 

***3.7 Study treatment.*** The standard treatment group will receive intramuscular treatment with 21,000 units equine antiserum (Viet Nam) or 3000 IU human antitoxin (CSL Behring) including a 0.05ml test dose (i.e. 75 units equine antitoxin or 12.5 IU human antitoxin). These are the recommended doses for treatment of tetanus. This equates to a total 14ml equine antitoxin and 12 ml human antitoxin. The intrathecal intervention group will receive 500 IU (total 2 ml) human tetanus antitoxin intrathecally. Both groups will receive this treatment as soon as possible after enrolment, with all antitoxin aimed to be given within 6 hours of admission. Patients who have been given a treatment dose of intramuscular antitoxin before admission will have the intramuscular injection omitted. The dose of 500 IU has been chosen on the basis of previous studies which have used between 250 and 1000 IU, but possible benefit of doses > 250 IU human antitoxin were noted in one meta-analysis ^20^. 250 IU doses have been used without reports of harm in neonates. Equally no difference in benefit was observed in a study comparing 200 and 1500 units of equine antitoxin ^14^.

Both equine and human preparations will be stored between 2 and 8°C. Prepared study packs will also be stored at this temperature until used. All receipt, transfers, dispensing, administration and return of study drug will be accounted for by the trial pharmacist. Study drug will be prescribed and administered by standard hospital and Oxford University Clinical Research Unit (OUCRU) procedures. Ward nurses trained in study procedures will be responsible for safe storage and in-hospital administration.

All other treatments will follow standard management of tetanus at the Hospital for Tropical Diseases. This will comprise of antibiotics, spasm control and cardiovascular modulation as current protocols dictate. Sedation will follow standard protocol: diazepam, following tracheostomy magnesium sulphate, then paralysis with pipecuronium.

***3.8 Blinding procedures.*** Investigators and attending doctors will be blinded to treatment allocation. Only trained and knowledgeable staff, trained in Good Clinical Practice and the protocol, and delegated in the delegation log will be able to perform study procedures. This will be as follows:

• The attending doctors will contact the independent study doctor (see below) when a patient has entered the study. The independent study doctor who does not have normal duties on the ICU nor responsibility for day-to-day care of tetanus patients will access the treatment pack and allocation to ascertain whether the additional intrathecal injection or a sham procedure is to be performed.
• The independent study doctor or a similarly independent study nurse will deliver the initial intramuscular injection of antitoxin, thus doctors and nurses treating patients will not be aware of initial intramuscular treatment.
• The intrathecal injection or sham procedure will be given by the independent doctor behind screens. The sham procedure will involve placing a dressing over the lumbar area in an identical way to the procedure after true lumbar puncture. In this way doctors and nurses treating the patient will not be aware of the intrathecal treatment. Patients will be asked not to discuss their given treatment with doctors or nurses caring for them.
• All patients will be kept in the supine position for 4 hours after intrathecal injection or sham procedure, in keeping with normal procedure following lumbar puncture. Supine position of tetanus patients has been shown to be non-inferior to semi-recumbent ^25^, therefore we do not think this is deleterious to patient care.
• No open record of the lumbar puncture procedure or study drug administered will be kept in the clinical notes. A sealed record of the intrathecal injection procedure will be placed in a sealed envelope in the patients notes to be opened if the attending clinicians feel necessary.
• Daily management decisions will be made by attending doctors (responsible for day-to-day patient care) following normal protocols and using defined criteria for mechanical ventilation.

***3.9 Baseline assessment.*** This will consist of basic clinical and demographic data, including SOFA score and Tetanus Severity Score. If a wound is present swabs will be taken for *Clostridium tetani* culture. This will provide supportive data for the diagnosis of tetanus. Bacterial DNA may be stored for later analysis.

Cerebrospinal fluid (CSF) that has been withdrawn for antitoxin administration will be stored for analysis of tetanus toxin and antitoxin concentration.

Residual samples from these analyses may be stored for use in future studies.

***3.10 Subsequent assessments.*** Whilst the patient is in ICU, daily treatment interventions (i.e. tracheostomy or mechanical ventilation), doses of predefined drugs, heart rate, blood pressure, respiratory rate, SpO _2_ and temperature will be recorded. Incidence of predefined hospital-acquired infection and new antibiotic prescriptions will be recorded.

Daily doses of diazepam will continue to be recorded after discharge from ICU until cessation of therapy.

Urinary catecholamines will be measured from a 24-hour collection taken on day 5.

• Criteria for tracheostomy are: laryngeal spasm or sputum retention; or requirement for muscle paralysis and mechanical ventilation due to uncontrolled spasm.

***3.11 Discontinuation of treatment and participation.*** If a patient or the representative, who has given consent on their behalf, chooses to discontinue trial treatment, they should be followed up (providing they are willing) and encouraged to follow the study procedures in lieu of withdrawing from the trial. If they do not wish to remain on trial follow-up, however, their decision will be respected and the patient will be withdrawn from the trial completely. This will be recorded on the OUTCOME Case Report Form (see Supplementary File 4). The reason for the patient withdrawing should be ascertained wherever possible. Prior to withdrawing completely from the trial, the patient will be invited to have assessments performed as appropriate for the final visit although they would be at liberty to refuse any or all individual components of the assessment.

In addition, the investigator may decide to stop the study intervention if they feel it would not be in the patient’s best interests. Patients will be followed as per protocol. Reasons for stopping the intervention would include, but are not limited to:

• Pregnancy
• Ineligibility (either arising during the study or retrospective having been overlooked at screening e.g. an alternative diagnosis to tetanus being confirmed)
• Significant non-compliance with treatment regimen or study requirements
• Allergic reaction to intramuscular injection
• Inability to administer intrathecal antitoxin

***3.12 Unblinding.*** Unblinding means revealing the identity of the study treatment (i.e. intrathecal or intramuscular only). Study treatment should only be unblinded if knowing the treatment that a patient has been allocated will result in a change in the patient’s management. The decision whether or not to unblind should be discussed with the Principal Investigator. Unblinded treatment allocation information will be available in opaque, tamper-proof envelopes held securely at each site and available at all times. A record of the lumbar puncture procedure will be available in a sealed envelope in the patients’ notes. The responsibility to approve unblinding will be assigned to dedicated site staff. Access to treatment allocation information should only be given with the approval of one of these dedicated staff. Unblinding will be documented in the Case Report Form. 

### 4 Adverse events

Study treatment will be discontinued for all patients displaying anaphylactoid reactions (i.e. if a patient displays anaphylactoid signs after either a test dose or intramuscular dose of antitoxin, then no further antitoxin will be given regardless of treatment allocation). The occurrence of such reactions is rare (<0.5%) and reported to be lower with human than equine antitoxin ^7^. Management of adverse events will follow normal care and current practice guidelines and a standard operating procedure. As these reactions occur irrespective of route of administration, treatment does not require unblinding.

***4.1 Safety reporting***

Definitions

*Adverse Event (AE)* - is any untoward medical event that occurs to a study participant during the course of the study whether or not that event is considered related to the study drug. An AE can, therefore, be any unfavorable and unintended sign (including an abnormal laboratory finding, for example), symptom, or disease temporally associated with the study drug, whether or not considered related to the study drug.

Stable chronic conditions, such as arthritis, which are present prior to clinical trial entry and do not worsen are not considered AEs and will be documented in the subject’s clinical chart as medical history. 

Clinical or laboratory events are considered AEs only if they occur after the first dose of study treatment (see below for reporting of AEs).

*Serious Adverse Event (SAE)* - An AE is considered to be "serious" if it results in one of the following outcomes

• Death
• Life-threatening event (the subject was at immediate risk of death at the time of the event; it does not refer to an event which hypothetically might have caused death if it were more severe)
• Inpatient hospitalization (new admissions) or prolongation of existing hospitalization (beyond what is expected for normal clinical care)
• Persistent or significant disability/incapacity (a substantial disruption of a person's ability to conduct normal life functions)
• Congenital anomaly/birth defect

Any undiagnosed pregnancy during which treatment occurred will be followed until outcome. Any congenital abnormality or birth defect will be recorded as a SAE.

*Unexpected Serious Adverse Events (USAE) -* are untoward medical events which fit one or more of the criteria for SAEs above and which are not considered a part of normal clinical progression of disease or an expected reaction to standard treatment therapy. Any event that becomes of concern to the investigators or study doctors during the course of the trial may be reported as a USAE.

***4.2 Assessment of AEs.*** AEs will be graded according to the Common Terminology Criteria for Adverse Events (CTCAE) definitions ^26^. In the event that an AE is not described within the CTCAE definitions, the following generic severity grading will be used:

• Grade 1 Mild: asymptomatic or mild symptoms; clinical or diagnostic observations only; intervention not indicated.
• Grade 2 Moderate: minimal, local or noninvasive intervention indicated; limiting age-appropriate instrumental Activities of Daily Living (ADL) ^a^.
• Grade 3 Severe or medically significant but not immediately life-threatening: hospitalization or prolongation of hospitalization indicated; disabling; limiting self-care ADL ^b^.
• Grade 4 Life-threatening consequences: urgent intervention indicated.

[Note: “Life-threatening” as a severity grade is not necessarily the same as “life-threatening” as a “serious” criterion used to define a SAE. The former is a “potential” threat to life and the latter is an “immediate” threat to life.]

A laboratory abnormality only needs to be recorded as a clinical AE if it is associated with an intervention. Intervention includes, but is not limited to, discontinuation of a current treatment, dose reduction/delay of a current treatment, or initiation of a specific treatment. In addition, any medically important laboratory abnormality may be reported as an AE at the discretion of the investigator. This would include a laboratory result for which there is no intervention but the abnormal value suggests disease or organ toxicity. Laboratory events will be graded according to CTCAE definitions.

If clinical sequelae are associated with a laboratory abnormality, the diagnosis or medical condition should be reported as the AE (e.g. renal failure, haematuria) - not the laboratory abnormality (e.g. elevated creatinine, urine RBC increase).

The relationship of each AE to the trial medication must be determined by a medically qualified individual according to the following definitions:

• Related: The AE follows a temporal sequence from trial medication administration, follows a known pattern of response for which no other explanation is present.
• Possibly related: The AE has a temporal relationship to the trial medication administration, does not follow a known pattern of response but cannot be attributed to another cause.
• Not related: The AE is probably produced by the participant’s clinical state or by other modes of therapy administered to the participant.

Patients will be under active surveillance for AEs. All AEs will be scored as not related, possibly related, or related to the study drug. The events listed below, and any others agreed between the treating doctor and Principal Investigator, are recognised adverse reactions to anti-toxin may be considered related to the trial intervention: chills, fever, myalgia, moderate back pain, arthralgia, local pain and redness at injection site, itching, rash, hypotension. Rarely anaphylactic reactions may occur.

In addition AEs related to lumbar puncture procedure will include headache, bloody tap, infection, vomiting, dysaesthesia, epidural, subdural or subarachnoid hemorrhage, post dural puncture, cerebral herniation.

AEs related to intra-muscular injection will include abscess formation, tissue fibrosis, contracture, haematoma, injury to blood vessels, bones and peripheral nerves.

***4.3 AE recording.*** All AEs which initiate after administration of the first dose of study drug and before discharge from ICU will be recorded in the Case Report Form. All events judged to be possibly related or related to the trial intervention will be followed to resolution.

***4.4 Regulatory reporting of AEs.*** As SEAs and mortality are common in tetanus, safety reporting will focus on events of potential relevance to the trial intervention. The following SAEs will be reported to the relevant ethics committee:

• All unexpected SAEs
• All SAEs judged to be related or possibly related to the trial intervention
• All deaths or life-threatening events

The above SAEs will be reported as soon as possible but within 24 hours of the time of acknowledgement of the SAE to the site ethics committee.

An initial written report will be sent as soon as possible but not later than 7 days from the acknowledgement of the event. The format and content of the initial report should follow the Viet Nam Ministry of Health Ethics Committee report template and include all information available at the time of reporting. A follow up report with complete details will be sent within 15 days since the time of acknowledgement if the initial report does not contain the details of event resolution.

All SAEs that do not meet criteria above will be included in the annual report to the Ministry of Health EC Viet Nam.

***4.5 Safety reporting and the Data Safety and Monitoring Board.*** An independent Data and Safety Monitoring Board will be established consisting of expert Vietnamese and international researchers and doctors, with the necessary clinical, research and statistical knowledge. The Data and Safety Monitoring Board will review the protocol and agree to a data review schedule and reporting requirements applicable before the study commences, with particular reference to the details of the interim review. A Data and Safety Monitoring Board charter will outline its responsibilities and how it will operate.

The DSMB will perform a safety review of data for the first 20 patients enrolled to the trial. This review will include unblinded summary tables of baseline characteristics, SAEs, AEs and event reports submitted to the DSMB. An analysis of overall clinical outcome will be performed. An annual safety review will be performed and, if deemed necessary, an additional safety review may be performed after the enrolment of 60 patients or other interval at the discretion of the Data and Safety Monitoring Board based on available data and ongoing reporting. All Data and Safety Monitoring Board reports will be sent to the responsible ethical committees including the site ethics committees, the Oxford Tropical Research Ethics Committee and the Viet Nam Ministry of Health ethics committee for consideration. Recruitment will continue during the Data and Safety Monitoring Board review period. 

As the dissemination of preliminary summary data could influence the subsequent conduct of the trial and introduce bias, access to interim data and results will be confidential and strictly limited to the Data and Safety Monitoring Board members. No results (except for the recommendation) will be communicated to the outside and/or the clinical investigators involved in the trial.

***4.6 Protocol violations.*** Protocol violations are events that contradict or omit protocol instructions and fulfil one or more of the following criteria: 1) the safety/welfare of one or more patients is put at risk by the non-compliance, 2) the integrity of study data is compromised by the violation.

Protocol violations must be reported to the sponsor as soon as possible. If the principal investigator or sponsor confirms that the violation poses a risk to patient safety/welfare or the integrity of study data, the protocol violation must be reported to the responsible hospital Ethics Committee.

### 5 Statistics

***5.1 Sample size justification.*** Data from the Hospital for Tropical Diseases in 2013 showed that 40% of patients with tetanus required ventilation (43 out of 110 in a 6 month period). However an estimated 4–6% of patients did not have generalized tetanus and would be excluded from study entry. Therefore we estimate that 45% of study subjects without intrathecal treatment will require mechanical ventilation in our study. The main comparison of this 2x2 factorial trial is the comparison between subjects receiving intrathecal human antitoxin versus those without intrathecal treatment. To detect an absolute risk reduction for mechanical ventilation due to intrathecal treatment by 17% (from 45% to 28%) with 80% power at the two-sided 5% significance level, 250 subjects are required. To account for some protocol violations and losses to follow-up, the trial will randomize a total of 272 patients (68 in each of the four treatment arms in the 2x2 factorial trial). Should the ventilation rate in the control arm be lower than anticipated, this samples size will also detect a reduction from 40% to 24% with 80% power.

Regarding the secondary endpoint of ICU stay, median (interquartile-range) duration of ICU stay from historic data of 111 tetanus admitted to the Hospital for Tropical Diseases was 13 ^4– 22^ days. Based on simulation and these historical data, 272 subjects will provide 88% power to detect a relative reduction in the ICU stay by 25% using the Cox proportional hazards model.

***5.2 Description of statistical methods.*** The primary analysis population for all analysis is the full analysis population containing all randomized patients. Patients will be analyzed according to their randomized arm (intention-to-treat). Analyses for the primary endpoint will be repeated on the per protocol population which excludes the following patients: patients not receiving the randomized intervention and other major violations of inclusion/exclusion criteria or study procedures. 

The primary outcome measure, requirement for mechanical ventilation during ICU stay, will be summarized as x/n (%) in each group and compared between the groups based on a logistic regression model with the two interventions (intrathecal treatment vs. sham procedure, human vs equine intramuscular treatment) as the only covariates without an interaction term. The main comparison is the comparison between intrathecal treatment vs. sham procedure, but the effect of the second randomized intervention will also be summarized as a secondary comparison. As odds ratios from logistic regression are somewhat difficult to interpret for a clinical audience, we will additionally summarize relative risk reductions between the groups based on a binary regression model with a log-link rather than the logit link function used in logistic regression. Interactions between the two interventions are not expected and interaction tests will have low power but we will nevertheless assess potential interactions informally through interaction plots and formally through a likelihood ratio test for an interaction in the logistic regression model. Should the interaction test reach formal significance, we will also perform pair-wise comparisons between the 4 treatment arms.

Potential heterogeneity of the treatment effect will be assessed based on appropriate interaction (likelihood ratio) tests and the following pre-defined sub-grouping variables:

• The Tetanus Severity Score ^27^ (calculated based on information available prior to randomization only) stratified according to tertiles of the observed TSS.
• Age: (≤70, 71-80, >80 years)
• Pre-existing medical conditions: moderate severe illness, severe illness defined according to ASA physical state scale
• Anti-toxin prior to admission

In addition, the primary endpoint will be modelled using a logistic regression model including the two interventions plus additional adjustment for the Tetanus Severity Score, age and pre-existing medical conditions.

Duration of hospital stay, duration of ICU stay, and 240 day mortality will be visualized using Kaplan-Meier curves for all 4 study arms and, additionally, for all patients with and without intrathecal treatment. Formal comparisons of these outcomes (and in-hospital mortality) will be based on Cox regression models with the two interventions as the only covariates using a similar analysis strategy as for the primary outcome described above. 

Other binary endpoints will be analysed in the same way as for the primary outcome. In addition, the rate of new antibiotics prescriptions, ventilator associated pneumonia and other predefined HAI, and the clinical syndrome of autonomic nervous system dysfunction will be modelled with a Poisson-regression model with the log-transformed duration of ICU stay included as an offset.

The cost–effectiveness analysis will be performed from the healthcare provider perspective, thus including direct costs resulting from the implementation of the intervention in the ICU and subsequent costs during the hospital stay, as well as any costs associated with re-admission during the 240 day follow-up. This will include the costs for standard tetanus treatment, the incremental cost of intrathecal human antitoxin administration, and those for any other diagnostics and treatments provided during ICU admission, including time spent on mechanical ventilation.

Prospectively collected data entered in the Case Report Forms from all patients included in the trial will be used for the cost analyses. The unit costs of intrathecal human antitoxin administration and those for mechanical ventilation will be estimated using a detailed micro costing approach. The cost per day of mechanical ventilation will account for the annualized cost of the equipment, maintenance, consumables and staff time.

If the intervention does improve patient survival at a higher cost, we will calculate the incremental cost per ICU survivor. This will be extended to estimating the cost per quality adjusted life year (QALY) gained using the survival data at 240-day-after enrolment follow-up and modelling the anticipated QALYs gained for these patients based on life expectancy and QALYs gained in ICU survivors in the region.

Statistical analysis will be carried out using the latest version of R.

### 6 Data

***6.1 Data collection and entry.*** Source documents will be generated during the study by the site ward and study staff. Source documents include all original recordings of observations or notations of clinical activities, and all reports and records necessary for the evaluation and reconstruction of the clinical trial. Source documents include, but are not limited to, the subject’s medical records, research case record forms (paper or electronic), laboratory reports, radiologist’s reports, progress notes, pharmacy records, and any other similar reports or records of procedures performed during the subject’s participation in the study. 

Access to applicable source documents is required for study purposes. The site investigators are responsible for maintaining any source documentation related to the study. Source documentation should support the data collected on the Case Report Form when the Case Report Form is not the original site of recording, or else the reason for the difference documented. Source documentation must be available for review or audit by the sponsor or designee and any applicable regulatory authorities.

Case Report Forms will be used as a data collection tool. Case Report Forms may be used as source documents if they are the primary data collection tool for specified data as documented in written standard operating procedures. The Site Investigators are responsible for maintaining accurate, complete and up-to-date records. These forms are to be completed on an ongoing basis during the course of the study by authorized individuals.

Corrections to paper Case Report Forms must be initialled and dated by the person making the correction and must not obliterate the original entry. All Case Report Forms should be reviewed by the designated study staff and signed as required with written or electronic signature, as appropriate.

Selected study members will be trained on how to enter all clinical data as source information from the Case Report Forms and from laboratory source documents into an internet-based computerized data entry system called CliRes hosted by OUCRU. Source documents and electronic data will be verified according to the Data Management Plan and Trial Monitoring Plan.

***6.2 Record retention.*** The investigator is responsible for retaining all essential data for at least 15 years after the completion of the trial. Original paper documents will be maintained for a minimum of 5 years and electronic documents retained thereafter. All stored records are to be kept secure and confidential.

### 7 Quality control and assurance

The study will be conducted in accordance with the current approved protocol, International Council for Harmonization Good Clinical Practice, relevant regulations and standard operating procedures.

Regular monitoring will be performed according to International Council for Harmonization Good Clinical Practice by OUCRU Clinical Trials Unit. Data, drugs, samples and procedures will be evaluated for compliance with the protocol, standard operating procedures, regulatory requirements and terms of ethical approval. Records will be verified for accuracy against source documents and physical inventory of drugs and samples.

### 8 Ethics

***8.1 Ethical and regulatory guidelines.*** The Principal Investigator will ensure that this study is conducted in accordance with the principles of the Declaration of Helsinki (Seoul 2008) and the terms of approval of the appropriate ethics committees. The Investigator will ensure that this study is conducted in full conformity with relevant regulations and with the International Council for Harmonization for Good Clinical Practice July 1996.

***8.2 Ethical review.*** This protocol and all associated informed consent forms has been approved by the Ethics Committee of the Hospital for Tropical Diseases (816/QD BVBND), Oxford Tropical Research Ethics Committee ^16, 17^ and the Viet Nam Ministry of Health (1122/QD-BYT). The investigators will submit and obtain, where necessary, approval from the above parties for all substantial amendments to the original approved documents.

***8.3 Risks and benefits.*** All patients will receive the best available standard-of-care in Vietnam. Side effects associated with the use of equine anti-toxin at the Hospital for Tropical Diseases are infrequent (< 1%). Human immunoglobulin is reported to be associated with an improved safety profile compared to equine preparations ^26^.

Risks of procedures involved in the study

Risk of intramuscular injection (all groups), which are minimal. All tetanus patients currently receive intramuscular injection of antitoxin; therefore there is no additional risk associated with participating in this study. Intramuscular injection of human antitoxin is reported to be associated with fewer adverse reactions than the current standard equine antitoxin.

The intrathecal intervention group will have the additional risk of lumbar puncture. The most serious complication that may be linked to lumbar puncture is cerebral herniation. This has never been reported in patients treated with intrathecal antitoxin in tetanus. This event is argued to be a result of associated raised intracranial pressure not the lumbar puncture procedure and as raised intracranial pressure does not occur in tetanus the risk of this event is extremely low (<0.001%). No lumbar punctures will be performed if the patient is suspected of having raised intracranial pressure or has a contra-indication to lumbar puncture. Other risks of lumbar puncture include infection (rare and currently < 0.1% at Hospital for Tropical Diseases), headache and venous puncture. Staff who will be performing the lumbar puncture are highly experienced clinicians who have performed this procedure >500 times.

Risks of intrathecal antitoxin use

Studies using intrathecal antitoxin report a low incidence of AEs. Most older studies used either equine antitoxin or human antitoxin preparations containing thimerosal preservative, which has been suggested to be responsible for many of the side effects. Only 1 published study of human tetanus immunoglobulin has reported the exact formulation used. In this study by Menon *et al.*, 41 patients were treated with TetGlob, a product containing thimerosal. One patient was reported to have mild learning difficulties and cerebral palsy on long-term follow up; a child aged of 11 months at the time of treatment, the authors felt this was most likely due to the age of the child and severity of disease. Studies in Africa have reported incidence of learning difficulties and cerebral palsy to be 20–40% in survivors of neonatal tetanus ^28, 29^ treated without intrathecal antitoxin.

Tetagam-P used in this study contains no preservatives or alcohol.

In clinical trials involving 947 patients treated with intrathecal antitoxin there were 3 cases of neonatal death. These consist of 2 cases of ‘apnea’ in Chugh *et al.’s* study in 1984 and 1 case of sudden death 1 hour after intrathecal injection of 250 units equine antitoxin/ and 6 ml hydrocortisone in a neonate. Unfortunately the paper by Chugh *et al.* is no longer available and further details about these deaths are not available and no details about the antitoxin are known. Other side effects reported in clinical trials are 5 cases of vomiting when using intrathecal antitoxin with preservative, and one post lumbar-puncture headache which was reported in 1 out of the 58 patients in the study by Miranda-Filhao *et al.* ^8^ and eliminated after administration of a non-steroidal anti-inflammatory agent. Mild headache was also reported in some patients during injection, but this was reduced by slow injection.

In a total of 390 patients treated with intrathecal antitoxin (mainly adults) where studies explicitly looked for and reported side effects, no serious side-effects were reported.

AEs in intrathecal antitoxin used outside of clinical trials

In published case series (total of 219 patients treated with intrathecal antitoxin), the only adverse effect reported was during long-term follow up of 9 out of 41 patients in one study where one patient was found to have mild mental retardation and cerebral palsy, which was thought to be due to the severity of disease and the patient’s young age. Similar AEs have been reported in neonatal tetanus patients not treated with intrathecal antitoxin ^29^.

In other published literature, Robert *et al.* reports of 2 cases of reversible paraplegia after high-dose of antitoxin, however it is not clear whether these were related to the high dose of antitoxin used (1500–2000 IU) or the preservatives used in preparations. Preparations contained both mercury and alcohol preservatives ^30^. Recent studies have used lower doses of preservative-free preparations without any reported of SAEs.

General points about intrathecal administration of drugs

Intrathecal drug delivery is currently approved in the UK for the treatment of chronic muscle spasticity (e.g. intrathecal baclofen injection/infusion) as well as management of cancer, chronic non-malignant or neuropathic pain (e.g. intrathecal morphine) chemotherapy treatment for lymphomatous meningitis (e.g. methotrexate, cytarabine) and adjuvant antibiotic therapy in bacterial meningitis and other CNS infections. Intrathecal formulations must be preservative-free as preservatives such as parabens and benzyl alcohol can cause arachnoiditis and nerve damage. Neurotoxicity is the main side effect of intrathecal drug delivery arising from unsuitable excipients, buffers, solubility enhancers and even the active drug itself. Ideally, intrathecal formulations should contain as few excipients as possible and the active drug must be screened for its propensity to cause neurotoxicity.

Tetagam-P

Tetagam-P is prepared as a pre-mixed syringe containing at least 95% human protein immunoglobulin with at least 250 IU antibodies to tetanus toxin as the active ingredient. Other ingredients are aminoacetic acid (glycine), sodium chloride, HCl or NaOH in small amounts for pH adjustment and water for injection. It is essentially sodium free with neutral pH. Tetagam-P contains no preservatives or alcohol. It comes as a pre-mixed solution minimizing error in re-constituting powder and potentially reducing the risk of introducing infection.

Studies in humans and animals, whilst small-scale, do not show any harm from the use of glycine within the CNS. As glycine is one of the important neurotransmitters within the CNS its use has been suggested to treat of chronic pain ^31, 32^.

The study by Menon *et al.* used ‘TetGlob’ intrathecally ^28^. This human immunoglobulin is similar in formulation to Tetagam-P (except TetGlob contains thimerosal) and is licensed for intrathecal use. We therefore believe that Tetagam-P is safer for intrathecal injection than TetGlob as it does not contain the thimerosal preservative. Tetagam-P is also produced by CSL Behring, a respected manufacturer of high quality immunoglobulin products and is therefore the best quality product available.

To ensure safety during this trial:

1. Sequential spaced enrolment of the 5 participants during a pilot phase will occur. All AEs will be monitored and reported as detailed above.
2. Only doctors experienced in lumbar punctures, trained in Good Clinical Practice and the protocol will perform the procedure
3. No patients with contra-indications to lumbar puncture will be enrolled
4. Injection will be made slowly and equivalent amount of CSF will be removed. Injections will be given with a single injection. No indwelling catheters will be used to prevent the risk of accidental injection of other drugs intrathecally or complications such as granulomas or infection.
5. Injections will be made after withdrawal of CSF to ensure no inadvertent intravascular injection.
6. Tetagam-P will be used as the intrathecal preparation. This contains no preservatives (including thimerosal or alcohol) but is otherwise similar to TetGLob, the preparation permitted for intrathecal use.
7. We have consulted clinical pharmacists about possible risks and contraindications for the use of intrathecal Tetagam in this study. All advice and recommendations have been taken into account in the design of this protocol.

Trial benefits

Against these risks, trial patients may benefit from receiving intrathecal antitoxin by experiencing less severe disease, lower mortality, reduced length of hospital stay, reduced complications such as hospital acquired infection, myocardial infarction, hypertension, hypotension and improved long-term outcome. In addition, all patients in the study will benefit from the careful observation and follow-up from enrolment, which will allow the complications of tetanus to be rapidly identified and managed.

The risks and benefits of participation will be communicated in two ways. First, all potential patients or their representatives will be given an informed consent form clearly listing the risks and benefits of the trial. Second, all potential patients (or their representatives) will be able to discuss participation with their physician who will be able to address questions not covered or arising from the patient information sheet.

Patients’ confidentiality will be maintained throughout the trial. Data submitted to OUCRU Clinical Trials Unit and samples sent to central testing facilities will be identified only by the trial number and patient initials.

***8.4 Expenses and benefits***

The study funding will cover the following costs:

• Study specific screening tests and procedures
• Diagnostic, treatment and hospital costs from enrolment to hospital discharge
• Hospital cost for patients readmitted after first discharge for AEs associated with the study treatment during the 240 day follow up.
• Study-related follow-up visits during the 240 day follow-up.
• Treatment of any AEs which are caused by study participation
• The study will not cover the cost of treating pre-existing diseases or those unrelated to study participation or the diagnosis and/or treatment of tetanus.

***8.5 Participant confidentiality.*** The trial staff will ensure that the participants’ anonymity is maintained. Participants will be identified only by initials and a participant identification number on the CSF, samples and any electronic database. All documents will be stored securely and only accessible by trial staff and authorised personnel.

### 9 Sample use and storage

Samples collected will be used for the purpose of this study as stated in the protocol and stored for future use in studies not yet conceived within Viet Nam or abroad. Consent will be obtained from subjects for genetic testing and for sample storage and/or shipment of specific samples to collaborating institutions for investigations that cannot be performed locally. Any proposed plans to use samples other than for those investigations detailed in this protocol will be submitted to the relevant ethics committees prior to any testing.

The participants will be identified only by a study specific participant number and/or code in any database. The name and any other identifying detail will NOT be included in any study data electronic file.

### 10 Finance and insurance

The conduct of this study is funded by the Wellcome Trust and sponsored by the University of Oxford. The University has a specialist insurance policy in place: - Newline Underwriting Management Ltd, at Lloyd’s of London – which would operate in the event of any participant suffering harm as a result of their involvement in the research.

### 11 Publication policy

The primary outcome data will be analysed and reported in a publication. The authors (and their respective positions in the author list) will be agreed prior to the start of the study in accordance with the guidelines of the International Committee of Medical Journal Editors.

### 12 Trial status

The trial is registered NCT02999815 clinicaltrials.gov. Recruitment began in August 2017. Expected length of recruitment is 3 years.

## Discussion

Tetanus is a vaccine preventable disease that remains a significant health problem mainly in remote or low-resource settings with limited access to modern intensive care facilities ^2, 6^. Currently in these settings mortality from tetanus remains high, and simple but effective interventions are very much needed. Due to the ubiquitous nature of *Clostridium tetani,* the causative organism *,* complete disease eradication is not possible and unvaccinated populations will continue to contract the disease throughout the word. As even in well-resourced settings, tetanus is associated with high healthcare costs, prolonged ICU stay and long-term disability; effective treatments that can shorten hospital stay and reduce healthcare costs are still needed. We are carrying out this study as we believe intrathecal antitoxin is an intervention with the potential to benefit all patients with tetanus and can be performed in most settings.

This is the first study of intrathecal antitoxin in Vietnam and we have focused on safety at all stages of this study. Prior to embarking on this randomized controlled trial, a pilot study was performed in which 5 patients were treated with open-label human antitoxin intramuscularly and intrathecally. These patients were recruited sequentially and new patients were only enrolled after previous patients were discharged, and clinicians and the trial Data and Safety Monitoring Board were satisfied it was safe to continue. The pilot study results, including all AEs were reported to the trial DSMB, Hospital for Tropical Diseases Ethics Committee and the Ministry of Health Viet Nam Ethics Committee and permission to proceed obtained before enrolment in the randomized controlled trial was commenced. Whilst previous studies have reported reassuringly few AEs associated with administration of intrathecal antitoxin, patients in this study will be followed carefully daily by study physicians and nurses for any evidence of AEs. Furthermore patients will be followed up 240 days after discharge from hospital.

This is only the second study of intrathecal antitoxin that has attempted to blind attending doctors from treatment. Study staff are not in any way involved in the care of the patients in the study. Staff delivering the study interventions work in a different department and only come to the ICU to deliver the intervention (in a screened off area). Staff collecting end-point data are also independent from those delivering care and are not employed within the ICU. Mortality rates from tetanus in our unit are low and we have chosen mechanical ventilation as a primary endpoint as this is a clinically important outcome. Not only do patients requiring mechanical ventilation have significantly increased costs, but in our setting they also are central to the transmission of hospital acquired infection and the growing problem of antimicrobial resistance. Despite having ventilation criteria, we feel that this endpoint still may be subject to bias and therefore have designed a blinded study. We have used secondary outcomes of drug use, duration of ventilation and the occurrence autonomic dysfunction as additional markers of disease severity. In addition, we have added health economic analysis. We therefore hope that this trial will provide high-quality evidence for the use of intrathecal antitoxin valuable to all settings.

## Ethics approval and consent to participate

For Ethics related to this trial see section 8. This study has been approved by the Ethics committees of the Ministry of Health, Viet Nam, Hospital for Tropical Diseases, Ho Chi Minh City and the Oxford Tropical Research Ethics Committee.

For consent related to this trial see section 3.4.

## Data availability

No data is associated with this article.

## Footnotes

^a^ Instrumental ADL refer to preparing meals, shopping for groceries or clothes, using the telephone, managing money, etc.

^b^ Self-care ADL refer to bathing, dressing and undressing, feeding self, using the toilet, taking medications, and not bedridden.

## Supplementary material

Supplementary File 1: SPIRIT checklist.

Supplementary File 2: SPIRIT study procedures.

Supplementary File 3: Informed consent/assent form.

Supplementary File 4: Case Record Form in Vietnamese and English.
