# Supplementary material for: Intrathecal Immunoglobulin for treatment of adult patients with tetanus: A randomized controlled 2x2 factorial trial
Source: Wellcome Open Res. 2018 Nov 5;3:58. Originally published 2018 May 16. [Version 2] doi: 10.12688/wellcomeopenres.14587.2 (PMC6372971; doi:10.12688/wellcomeopenres.14587.2)
Supplement: Supplementary file 3 [file wellcomeopenres-3-16235-s0002.tgz › c139bf98-d592-44a4-a7ae-299abdba7efe.docx]

Participant Information sheet

**Intrathecal human Immunoglobulin for treatment of adult patients with tetanus: a randomized controlled 2x2 factorial trial**

Short title: Spinal injection of human immunoglobulins for treatment of tetanus.

***Note: If you are a parent or guardian of a child below 18 years old, please read "you" as "your child" and “your” as “your child’s”.***

You are being invited to take part in a research study of adults with tetanus. Whether or not you participate is your choice, and either way you will be given the best available treatment for your illness. Please read this information sheet carefully or have someone read it to you. You will be given a copy of this form to keep. Please ask the study staff to explain any information that you are not sure about.

**What is the reason for doing the study?**

Tetanus can be a severe disease and people with tetanus need to be treated in the intensive care unit for many weeks. Because of the severe muscle spasms the doctors may need use a machine to help you breathe and tetanus also causes problems with your heart and blood pressure.

Normal treatment of tetanus includes giving you an injection of antitoxin into your muscle but some people have tried giving the injection by spinal injection and reported that it may work better – making the tetanus much less severe. This means that patients may not need such expensive treatment (such as machine breathing) and may be able to go home much sooner. It may also mean fewer patients will die from tetanus. However at the moment it is not clear if the new treatment really is better than standard treatment and we need to carry out a careful trial to find this out.

We would like to see if adding spinal injection to muscle injection of antitoxin makes people get better from tetanus faster and stop them needed breathing support. We will also compare two types of antitoxin: the one currently used in Vietnam (made originally from horses) and one that many people believe causes less side effects (made from humans). The human antitoxin is commonly used in many countries in the world and is recommended by many organisations such as the World Health Organisation but it is not yet available in Vietnam.

This study will include 272 patients and is organized by the Hospital for Tropical Diseases and Oxford University. The study has been approved by the ethical committees of both institutions and the Viet Nam Ministry of Health.

**What will happen if you participate in the study?**

Whether or not you join the study, you will receive the standard treatment for tetanus including blood tests, and intensive care unit treatment.

If you agree to participate in the study you will be given one of the four following treatments:

1. Antitoxin injection into muscle with (normal) horse antitoxin
2. Antitoxin injection into muscle with human antitoxin
3. Antitoxin injection into muscle with (normal) horse antitoxin and spinal injection of human antitoxin
4. Antitoxin injection into muscle with human antitoxin and spinal injection of human antitoxin

You have an equal chance of getting each of the four treatments and which you get will be assigned by chance. The doctors treating you will not know which treatment you receive because the injections will be given by doctors from another department. These doctors are very experienced in spinal injections and have undergone special training for this study. The reason for doing this is that it allows the results of the study to be judged fairly to get the most reliable result. IF you do not receive the spinal injection the doctors will put a small dressing on your back to look as if you had the injection. If you have already had a full dose injection into muscle then this part of the treatment will not be given.

After this you will be treated as normal during your hospital stay. We will also telephone 240 days after enrolment to check how you are.

Patients enrolled to the study will have some additional lab tests:

- Spinal fluid test (in those having lumbar puncture): we will check this to see if the antitoxin you had by muscle injection has got to the spine. You will only have this test if you have the spinal injection and it involves analysing the small amount of fluid that we remove before we give the injection.
- Urine test: we will collect the urine you pass over a 24-hour period to check for signs of tetanus. This urine is being collected by the doctors anyway as part of your treatment.
- Bacteria culture test to help confirm the cause and site of the tetanus infection

**What else happens to the samples taken?**

Samples taken will help answer the questions asked by the study. If there is any spinal fluid (in those having lumbar puncture) remaining after these tests have been done it may be stored for future research on infectious diseases. Bacteria grown from wounds, may also be stored. This future research may include DNA testing and could be done outside of Viet Nam. The tests will be done anonymously (no one doing the tests will be able to identify you) and you will not be given the results of these tests. You can choose if you agree to this or not by marking the last page of this form.

# What are the possible risks of the study?

Studies that have used the spinal injection of tetanus toxin report very few side effects: the commonest are headache and vomiting in a few (<10) patients out of hundreds who have received this treatment.

In other diseases spinal injections have very occasionally been associated with more serious complications. The most common risks (affecting 1-10% of patients) include:

- Shooting pain down the legs at the time of the procedure which usually settles when the needle is removed.

Rare risks and complications (less than 1% of patients) for patients with other disease include:

- Bleeding at the site of needle insertion or into the spinal canal can be immediate or delayed. This is often harmless but may cause leg problems.
- Leg weakness or numbness which usually lasts only a short time, although very rarely can be permanent

Even more rare are:

- Infection in the bones of the back or the spinal fluid. It is very rare but death from meningitis can occur.
- Brain Herniation (Movement of the brain) is an exceedingly rare condition that can lead to death or severe disability. This occurs in patients with diseases affecting the pressure of fluid around the brain. Tetanus does not alter the brain pressure therefore we believe this complication is extremely unlikely.

In the unlikely event that your health is worse as a result of this study, the study organizers have insurance to cover your care.

**What are the possible benefits of the study?**

If you participate in the study you may receive treatment (by spinal injection) that is better than the current standard treatment and you may recover faster. The study will cover your hospital costs for treatment or visits related to the study procedures from the time you enroll.

Participants in this study will have additional medical monitoring by study doctors who will follow your progress carefully. Any problems they notice will be treated appropriately.

**Do I have to participate?**

Being in a research study is your decision. If you do not want to be in the study or if at any time during the study you decide to stop participating, the doctors will respect your decision. In both cases you will receive standard medical treatment.

The research doctors can withdraw you from the study if they think your health is placed at risk by participating. The sponsor or the ethics committees can also stop the study if they consider it necessary.

If you agree to be in the study, you will be responsible for following the study schedule of tests and taking your drugs.

**Will anyone know that I am participating in this study?**

All information about you will be kept confidential. Your medical records will be reviewed in strict confidence by those who are working on this study and may also be reviewed by the ethics committees and health authorities reviewing the study. Your name will not be used on any of the study documents or on the stored samples or in any reports or publications about this study.

# What if I have more questions?

You are encouraged to ask any questions related to this study during the time of participation. If you have any questions about this program, its procedures, risks and benefits, or alternatives please call Dr. Huynh Thi Loan at 090 8245933 .

If you have any questions about your rights as a subject in this study, you may want to talk to Dr. Huynh Thi Loan , or if you want to speak to someone outside of the program you may contact the Ethics Committee at the Hospital For Tropical Diseases at 08 3923 5904 .

**INFORMED CONSENT FORM**

**Intrathecal human Immunoglobulin for treatment of adult patients with tetanus: a randomized controlled 2x2 factorial trial**

Short title: Spinal injection of human immunoglobulins for treatment of tetanus.

- I have read the information given to me and freely agree to be in this study. I also have had a chance to discuss it with the study staff.
- I have been told about the risks and benefits. I got answers that I could understand to all my questions.
- I agree that my samples will be tested for research purposes and that this will include tests on my DNA
- I agree that testing on some samples will be done outside of Viet Nam.
- I understand that I can withdraw from the study at any time. If I stop the study, it will **not** affect my future care. If I decide to stop the study, I agree that the information collected up to the point when I stop, may continue to be used.
- Please check the box if you AGREE to the storage and future use of your samples:

🞏 I AGREE that the samples taken can be stored for other research studies about infectious diseases and these studies may be done outside of Viet Nam.

🞏 I AGREE that the samples taken can be stored for genetic testing and these tests may be done outside of Viet Nam.

**Participant:**

By signing my name here, I confirm what is written above.

| x__________________ | x___________________ | ___/____/_____ |
| --- | --- | --- |
| Participant Signature | Print Name | Date of Signature |

**OR** – If someone else gives consent on behalf of the participant:

| Patient’s name: | x __________________________________________________ | | |
| --- | --- | --- | --- |
| Signature of Person Giving Consent:  x_______________ | Print Name:  x_______________________ | Relationship to Participant:  x__________ | Date of Signature:  ____/____/____ |

**Investigator/Designee**:

I, the undersigned, have fully explained the relevant information of this study to the person named above and will provide her/him with a copy of this signed and dated informed consent form.

| x___________________ | x______________________________ | ___/____/_____ |
| --- | --- | --- |
| Investigator/Designee Signature | Print Name | Date of Signature |

**Witness:** (*If the person giving consent cannot read the form themselves, a witness must be present and sign here)*

I was present throughout the entire informed consent process with the participant. This form was read accurately to the volunteer, all questions from the volunteer were answered and the volunteer has agreed to take part in the research.

| x_______________ | x______________________________ | ____/____/_____ |
| --- | --- | --- |
| Witness Signature | Print Name | Date of Signature |

**ASSENT FORM**

**Intrathecal human Immunoglobulin for treatment of adult patients with tetanus: a randomized controlled 2x2 factorial trial**

Short title: Spinal injection of human immunoglobulins for treatment of tetanus.

- I have read the information given to me and freely agree to be in this study. I also have had a chance to discuss it with the study staff.
- I have been told about the risks and benefits. I got answers that I could understand to all my questions.
- I agree that my samples will be tested for research purposes and that this will include tests on my DNA
- I agree that testing on some samples will be done outside of Viet Nam.
- I understand that I can withdraw from the study at any time. If I stop the study, it will **not** affect my future care. If I decide to stop the study, I agree that the information collected up to the point when I stop, may continue to be used.
- Please check the box if you AGREE to the storage and future use of your samples:

🞏 I AGREE that the samples taken can be stored for other research studies about infectious diseases and these studies may be done outside of Viet Nam.

🞏 I AGREE that the samples taken can be stored for genetic testing and these tests may be done outside of Viet Nam.

**Participant:**

By signing my name here, I confirm what is written above.

| x__________________ | x_____________________________ | ____/____/____ |
| --- | --- | --- |
| Participant Signature | Print Name | Date of Signature |

**Witness**: *(If the person giving consent cannot read the form themselves, a witness must be present and sign here)*

I was present throughout the entire assent process with the participant. This form was read accurately to the volunteer, all questions from the volunteer were answered and the volunteer has agreed to take part in the research.

| x_______________ | x______________________________ | ____/____/_____ |
| --- | --- | --- |
| Witness Signature | Print Name | Date of Signature |

**Investigator/Designee**:

I, the undersigned, have fully explained the relevant information of this study to the person named above and will provide her/him with a copy of this signed and dated informed consent form.

| x___________________ | x______________________________ | ____/____/_____ |
| --- | --- | --- |
| Investigator/Designee Signature | Print Name | Date of Signature |
