# Supplementary material for: Intrathecal Immunoglobulin for treatment of adult patients with tetanus: A randomized controlled 2x2 factorial trial
Source: Wellcome Open Res. 2018 Nov 5;3:58. Originally published 2018 May 16. [Version 2] doi: 10.12688/wellcomeopenres.14587.2 (PMC6372971; doi:10.12688/wellcomeopenres.14587.2)
Supplement: Supplementary file 2 [file wellcomeopenres-3-16235-s0001.tgz › 4873a9b9-0674-4630-9c79-7cda5229a70d.doc]

Supplementary File 2. SPIRIT study procedures

|  | **STUDY PERIOD** | | | | | | | |
| --- | --- | --- | --- | --- | --- | --- | --- | --- |
|  | **Enrolment** | **Allocation** | **Post-allocation** | | | | | **Close-out** |
| **TIMEPOINT**** | ***Admission to ICU*** | **0** | ***Day1*** | ***Day 2-4*** | ***Day 5*** | ***Day 6 +*** | ***Hospital discharge*** | ***270 days*** |
| **ENROLMENT:** |  |  |  |  |  |  |  |  |
| **Eligibility screen** | X |  |  |  |  |  |  |  |
| **Informed consent** | X |  |  |  |  |  |  |  |
| ***[List other procedures]*** |  |  |  |  |  |  |  |  |
| **Allocation** |  | X |  |  |  |  |  |  |
| **INTERVENTIONS:** |  |  |  |  |  |  |  |  |
| ***[Intramuscular antitoxin]*** |  |  | X |  |  |  |  |  |
| ***[Intrathecal antitoxin/ sham procedure]*** |  |  | X |  |  |  |  |  |
| **ASSESSMENTS:** |  |  |  |  |  |  |  |  |
| ***Eligibility assessment*** | X | X |  |  |  |  |  |  |
| ***Baseline clinical data*** |  |  | X |  |  |  |  |  |
| ***Daily assessment for AEs, Endpoints*** |  |  |  | X | X | X | X |  |
| ***Urine catecholamines*** |  |  |  |  | X |  |  |  |
| ***Modified Rankin score*** |  |  |  |  |  |  |  | X |
