# Supplementary material for: Intrathecal Immunoglobulin for treatment of adult patients with tetanus: A randomized controlled 2x2 factorial trial
Source: Wellcome Open Res. 2018 Nov 5;3:58. Originally published 2018 May 16. [Version 2] doi: 10.12688/wellcomeopenres.14587.2 (PMC6372971; doi:10.12688/wellcomeopenres.14587.2)
Supplement: Supplementary file 4 [file wellcomeopenres-3-16235-s0003.tgz › a2a7b4a8-9228-4f55-a7ba-e03ddc34bd80.zip!/Supplementary File 4/03TS CRF Version 6 English.docx]

| 1. Patient’s name: [__________________________________________________]* | |
| --- | --- |
| 2. Year of birth: [___][___][___][___] or Age [___][___] years | |
| 3. Sex: ⬜ Male ⬜ Female | |
| **Inclusion Criteria (All answer has to be YES for patients in the study)** | |
| 4. Is the patient >=16 years old? | ⬜ YES ⬜ No |
| 5. Does the patient have generalized tetanus? | ⬜ YES ⬜ No |
| 6. Did the patient or their representative consent to participate in the study | ⬜ YES ⬜ No |
| **Exclusion Criteria (All answer has to be NO for patients enrolled in the study)** | |
| 7. Has the patient received antitoxin this episode | ⬜ YES ⬜ No |
| 8. Has the patient contraindication to antitoxin | ⬜ YES ⬜ No |
| 9. Has the patient contraindication to lumbar puncture | ⬜ YES ⬜ No |
| 10. Is the patient already receiving mechanical ventilation or expected to before intrathecal injection can be given | ⬜ YES ⬜ No |
| 11. Is the patient pregnant test given ⬜ | ⬜ YES ⬜ No |

***If the patient is not eligible or refuses consent STOP HERE. If patient enrolled, continue***

12. Did the patient/ relative consent to save samples	⬜ Yes	⬜ No

13. Patient’s/ relative’s Phone number:	[_________________________] *

Other phone number (if any)[:	[_________________________](Tel:[_________________________)]	⬜ NA

14. Current place of living: District :	[_______________________]

Province:	[_________________________________]

15. Date & time of randomisation:[___][___]/[___][___]/[___][___] at [___][___]:[___][___]

*dd	mm*	yy	*use 24 Hr clock*

| 1. **Hospital Number:** [___][___][___][___][___][___][___][___][___][___][___][___] | | | | | |  |
| --- | --- | --- | --- | --- | --- | --- |
| 2. **Date and time of admission to this hospital:**   [___][___]/[___][___]/[___][___] at [___][___]:[___][___]  *dd mm* yy *use 24 Hr clock* | | | | | |  |
| 3. **Source**: ⬜ Community ⬜ Other hospital   *If other hospital date of first admission there:* [___][___]/[___][___]/[___][___]  *dd mm* yy | | | | | |  |
| 4. **Date & time of admission to ICU at this hospital**:   [___][___]/[___][___]/[___][___] at [___][___]:[___][___]  *dd mm* yy *use 24 Hr clock* | | | | | |  |
| 1. **Date of this ICU discharge:** [___][___]/[___][___]/[___][___] (*dd/mm/*yy)   Total length of ICU stay [___][___][___] days | | | | | |  |
| 2. **Date of hospital discharge**: [___][___]/[___][___]/[___][___] (*dd/mm/*yy) | | | | | |  |
| 3. **Final outcome**: ⬜ Death ⬜ Home/transfer to Die ⬜ Hospital transfer ⬜ Home | | | | | |  |
| 5. **Self reported comorbidities prior to admission.** *(fill in all rows)* | | | | | |  |
| Hypertension | | ⬜ Yes ⬜ No ⬜ NA |  | |  |  |
| Myocardial infarction | | ⬜ Yes ⬜ No ⬜ NA | Congestive cardiac failure (GradeI-III) | | ⬜ Yes ⬜ No ⬜ NA |  |
| Angina on minimal exertion/rest pain | | ⬜ Yes ⬜ No ⬜ NA | Congestive cardiac failure Grade IV | | ⬜ Yes ⬜ No ⬜ NA |  |
| Peripheral vascular disease | | ⬜ Yes ⬜ No ⬜ NA | Cerebrovascular disease | | ⬜ Yes ⬜ No ⬜ NA |  |
| Chronic pulmonary disease | | ⬜ Yes ⬜ No ⬜ NA | Severe respiratory deficiency | | ⬜ Yes ⬜ No ⬜ NA |  |
| Connective tissue disease | | ⬜ Yes ⬜ No ⬜ NA | Peptic ulcer disease | | ⬜ Yes ⬜ No ⬜ NA |  |
| Mild liver disease | | ⬜ Yes ⬜ No ⬜ NA | Diabetes | | ⬜ Yes ⬜ No ⬜ NA |  |
| Hemiplegia or paraplegia | | ⬜ Yes ⬜ No ⬜ NA | Moderate/severe kidney disease | | ⬜ Yes ⬜ No ⬜ NA |  |
| Diabetes with chronic complications | | ⬜ Yes ⬜ No ⬜ NA | Any malignancy | | ⬜ Yes ⬜ No ⬜ NA |  |
| Moderate or severe liver disease | | ⬜ Yes ⬜ No ⬜ NA | Metastatic solid tumour | | ⬜ Yes ⬜ No ⬜ NA |  |
| AIDS | | ⬜ Yes ⬜ No ⬜ NA | Dementia | | ⬜ Yes ⬜ No ⬜ NA |  |
| Other 1 (specify) | | ____________________ | Other 2 | | __________________ |  |
| Elective surgery within last 30 days ⬜ Yes ⬜ No ⬜ NA | | | | | |  |
| Emergency surgery within last 30 days ⬜ Yes ⬜ No ⬜ NA | | | | | |  |
| 6. Time from first symptom to hospital admission | | | | [__\|__] days | |  |
| 7. Incubation period [__\|__] days N/A ⭘ | | | | Period of onset [__\|__\|__] hours | |  |
| 8. Wound: Internal or injection ⭘ | | | | Other (or unknown) ⭘ | |  |
| 9. Difficulty breathing on admission to hospital | | | | Yes ⭘ No ⭘ | |  |
| 10. Weight: \|__\|__\|__] Kg | | | | height \|__\|__\|__] cm | |  |
| 11. ABLETT score | ⭘ I *(no spasm)* ⭘ II *(spasm not interfering with respiration)*  ⭘ III *(spasm interfering with respiration* ⭘ IV *(III plus ANSD)* | | | | | |
| 12. ASA | Fit and well ⭘  Minor illness or injury ⭘  Moderately severe disease ⭘  Severe illness not immediately life-threatening ⭘  Life-threatening illness ⭘ | | | | | |
| 13. Chỉ số SOFA/ APACHE/ TSS | Worst value in 1st 24 hours ICU  Highest temperature [__\|__\|.\|__]°C GCS [__\|**__]** RR [__\|__]_bpm_  FiO_2_ [__\|__\|% SpO_2_ [__\|__\|% PaO_2_ [__\|__\|__]mmHg pH [__\|. [__\|__]  Plt [__\|__\|__]10^3^/mm^3^ Wbc [__\|__\|__]10^9^/mm^3^ HCT [__\|__]%  Highest HR [__\|__\|__] bpm Lowest HR [__\|__\|__]bpm  Highest SBP [__\|__\|__] mmHg  Worst MAP [__\|__\|__]/[__\|__\|__]mmHg  Vasopressors: No ⭘ Có ⭘ If yes name …………………… dose …….. µg/kg/min  Bilirubin [__\|__\|__]mg/dL Na [__\|__\|__]mEq/L K [**__].**[__\|**__]**mEq/L  Creatinine [__\|__\|__] µmol/L Urine output [__\|__\|__\|__]mL/24hr  Acute renal failure Yes⭘ No ⭘ Immunocompromised Yes⭘ No ⭘ | | | | | |

| 14. Date IM antitoxin injection | [__\|__]/[__\|__]/[__\|__] | [__\|__]:[__\|__] |
| --- | --- | --- |
| IM antitoxin in previous hospital? | ⭘ Yes ⭘ No |  |
| 15. Date IT procedure | [__\|__]/[__\|__]/[__\|__] | [__\|__]:[__\|__] |
| 16. IM procedure complications | ⭘ Yes ⭘ No |  |
| If yes details: |  |  |
| 17. IT procedure complications | ⭘ Yes ⭘ No |  |
| If yes details: |  |  |

1. **Tracheostomy performed?:** ⬜ Yes	⬜ No	⬜ NA. ***If yes:***

Date & time of tracheostomy: [___][___]/[___][___]/[___][___] at [___][___]:[___][___]

*dd	mm*	yy	*use 24 Hr clock*

*Indication*

laryngeal spam	⬜

sputum retention	⬜

requirement for muscle paralysis and mechanical ventilation	⬜

2. **Date tracheostomy removed:**

Episode 1: date & time: [___][___]/[___][___]/[___][___] at [___][___]:[___][___]

*dd	mm*	yy	*use 24 Hr clock*

**More than one episode?** ⬜ Yes	⬜ No	*add supplementary sheets if >1 episode*

3. **Ventilation started:** ⬜ Yes ⬜ No [___][___]/[___][___]/[___][___] at [___][___]:[___][___]

*dd	mm*	yy	*use 24 Hr clock*

*Indication*

SaO_2_ <90%	⬜

PaO_2_/Fi0_2_ <250	⬜

excessive spasms necessitating muscle paralysis	⬜

4. **Date ventilation stopped:**

Episode 1: date & time: [___][___]/[___][___]/[___][___] at [___][___]:[___][___]

*dd	mm*	yy	*use 24 Hr clock*

Was the ventilation restarted? ⬜ Yes	⬜ No	⬜ NA. ***If yes:***

Episode 2 date & time: [___][___]/[___][___]/[___][___]at [___][___]:[___][___]

*dd	mm*	yy	*use 24 Hr clock*

When was ventilation stopped?

Episode 2 date & time: [___][___]/[___][___]/[___][___]at [___][___]:[___][___]

*dd	mm*	yy	*use 24 Hr clock*

**More than one episode?** ⬜ Yes	⬜ No	*add supplementary sheets if >1 episode*

1. **Ventilator Associated Pneumonia (VAP)	Date** (dd/mm/yy) **___/____/____**

Patient intubated and ventilated for at least 48 hours with 2 of the following:

+ Temperature > 38°C or < 36°C	☐ Yes ☐ No

+ Wbc < 4.0×10^9^/L or ≥ 12×10^9^/L	☐ Yes ☐ No

+ Purulent secretions	☐ Yes ☐ No

+ New infiltrations or deterioration of chest X-ray	☐ Yes ☐ No

**VAP**	☐ Yes ☐ No

Microbiologically confirmed VAP:

+ ETA culture > 10^5^ cfu/ml	☐ Yes ☐ No

2. **Autonomic Nervous System Dysfunction ANSD Date** (dd/mm/yy) **___/____/____**

Patient has at least 3 of the following:

+ HR > 100 bpm	☐ Yes ☐ No

+ High BP HA with SBP > 140 mmHg	☐ Yes ☐ No

+ Low BP with SBP < 60 mmHg	☐ Yes ☐ No

+ Temperature > 38°C	☐ Yes ☐ No

+ Fluctuating BP (High or low)	☐ Yes ☐ No

**ANSD**	☐ Yes ☐ No

3. **Pressure ulcer	Day of disease ..........................**

4. **GI Bleeding	Day of disease ..........................**

5. **Other** ☐ Yes ☐ No **.............................................................................................................**

| 1. **Date** (dd/mm/yy) | **____/____/____** | **____/____/____** | **____/____/____** | **____/____/____** | **____/____/____** | **____/____/____** | **____/____/____** |
| --- | --- | --- | --- | --- | --- | --- | --- |
| 2. **Temp. max** |  |  |  |  |  |  |  |
| 3. **SBP max/min** |  |  |  |  |  |  |  |
| 4. **HR max/min** |  |  |  |  |  |  |  |
| 5. **Midazolam IM** |  |  |  |  |  |  |  |
| 6. **Diazepam IM** |  |  |  |  |  |  |  |
| 7. **Oral Diazepam** |  |  |  |  |  |  |  |
| 8. **Pipecuronium** |  |  |  |  |  |  |  |
| 9. **Magnesium** |  |  |  |  |  |  |  |
| 10. **Fentanyl** |  |  |  |  |  |  |  |
| 11. **Vasopressors** |  |  |  |  |  |  |  |
| 12. **Ventilation** |  |  |  |  |  |  |  |
| 13. **ANSD** |  |  |  |  |  |  |  |
| 14. **Antibiotics +/-** |  |  |  |  |  |  |  |
| 15. **Indication** | O Tetanus  O VAP  O UTI  O BSI  O Other  ……………… | O Tetanus  O VAP  O UTI  O BSI  O Other  ……………… | O Tetanus  O VAP  O UTI  O BSI  O Other  ……………… | O Tetanus  O VAP  O UTI  O BSI  O Other  ……………… | O Tetanus  O VAP  O UTI  O BSI  O Other  ……………… | O Tetanus  O VAP  O UTI  O BSI  O Other  ……………… | O Tetanus  O VAP  O UTI  O BSI  O Other  ……………… |
| 16. **AE/SAE/USAE** |  |  |  |  |  |  |  |
| 17. **Other complications** |  |  |  |  |  |  |  |

1. **Patient completed?**

⬜ Yes

⬜ No, withdrew consent

⬜ No, withdrawn by doctor

⬜ No, died before randomization

⬜ No

⬜ No

*If no, date of withdrawal:* [___][___]/[___][___]/[___][___](*dd/mm/*yy)

2. **Total cost of ICU stay:**

[___] [___][___][___] [___][___][___] [___][___][___] Vietnamese Dong

3. **Cost of hospital stay**

[___] [___][___][___] [___][___][___] [___][___][___] Vietnamese Dong

1. **Day 240 follow up:	Date:**[___][___]/[___][___]/[___][___] (*dd/mm/*yy)
2. **Did successfully contact the patient:** ⬜ Yes	⬜ No. If no, specify the reason:

**_____________________________________________________________**

3. **Patient’s current status:**

⬜ Died. Date of death: :[___][___]/[___][___]/[___][___] (dd/mm/yy)

### [Causes of Death](http://www.cdc.gov/nchs/fastats/leading-causes-of-death.htm): ___________________________________________________

If the patient is alive

⬜ Home or relatives home	⬜ this hospital	⬜ another hospital

4. **Is the patient back at work?**

⬜ Yes to work as before

⬜ Part time or reduced hours

⬜ Not at all

5. **Any neurological problems?**

⬜ Weakness

⬜ Residual stiffness

⬜ Sensation abnormalities

⬜ Other ………………………………………………………………………………………………………………..

6. **Rankin Scale**

⬜ 0 No symptoms.

⬜ 1 No significant disability. Able to carry out all usual activities, despite some symptoms.

⬜ 2 Slight disability. Able to look after own affairs without assistance, but unable to carry out all previous activities.

⬜ 3 Moderate disability. Requires some help, but able to walk unassisted.

⬜ 4 Moderately severe disability. Unable to attend to own bodily needs without assistance, and unable to walk unassisted.

⬜ 5 Severe disability. Requires constant nursing care and attention, bedridden, incontinent.

⬜ 6 Dead

7. **Any other problems**
