# Supplementary material for: Intrathecal Immunoglobulin for treatment of adult patients with tetanus: A randomized controlled 2x2 factorial trial
Source: Wellcome Open Res. 2018 Nov 5;3:58. Originally published 2018 May 16. [Version 2] doi: 10.12688/wellcomeopenres.14587.2 (PMC6372971; doi:10.12688/wellcomeopenres.14587.2)
Supplement: Supplementary file 4 [file wellcomeopenres-3-16235-s0003.tgz › a2a7b4a8-9228-4f55-a7ba-e03ddc34bd80.zip!/Supplementary File 4/03TS CRF Vietnamese.docx]

| 1. Tên bệnh nhân: [__________________________________________________]* | |
| --- | --- |
| 2. Năm sinh: [___][___][___][___] hoặc tuổi: [___][___] (năm) | |
| 3. Giới: ⬜ Nam ⬜ Nữ | |
| **Tiêu chuẩn chọn vào (Tất cả các câu trả lời phải là“có” để bệnh nhân có thể được thu tuyển)** | |
| 4. Bệnh nhân ≥ 16 tuổi | ⬜ CÓ ⬜ KHÔNG |
| 5. Bệnh nhân có dược chân đoán là uốn ván | ⬜ CÓ ⬜ KHÔNG |
| 6. Bệnh nhân/người đại diện của họ có đồng ý tham gia nghiên cứu không? | ⬜ CÓ ⬜ KHÔNG |
| **Tiêu chuẩn loại trừ (Tất cả các câu trả lời “không” của bệnh nhân có thể được thu tuyển)** | |
| 7. Đã sử dụng SAT trước đó | ⬜ CÓ ⬜ KHÔNG |
| 8. Chống chỉ định sử dụng SAT hay HTIG | ⬜ CÓ ⬜ KHÔNG |
| 9. Chống chỉ định chọc dò dịch não tủy | ⬜ CÓ ⬜ KHÔNG |
| 10. Đã thở máy hoặc dự kiến thở máy trước khi được tiêm kênh tủy | ⬜ CÓ ⬜ KHÔNG |
| 11. Có thai Test ⬜ | ⬜ CÓ ⬜ KHÔNG |
| **Chấp thuận tham gia (Tất cả các câu trả lời phải là“có” để bệnh nhân có thể được thu tuyển)** | |
|  | |

***Nếu không đủ tiêu chuẩn hoặc bệnh nhân từ chối, DỪNG PHIẾU. Nếu được thu tuyển:***

12. Bệnh nhân/ thân nhân có đồng ý cho lưu mẫu bệnh phẩm không?	⬜ CÓ ⬜ KHÔNG

13. Số điện thoại của bệnh nhân/ thân nhân	[_________________________]

Số điện khác (nếu có)[:	[_________________________](Tel:[_________________________)]

14. Nơi ở hiện tại: Quận/huyện:	[_______________________]

Tỉnh	[_________________________________]

15. Ngày giờ phân nhóm ngẫu nhiên:[___][___]/[___][___]/[___][___] at [___][___]:[___][___]

*ngày	tháng	năm	Dùng định dạng 24 giờ*

| 1. **Mã bệnh án :** [___][___]. [___][___][___][___][___] | | | | | | | | | | | |
| --- | --- | --- | --- | --- | --- | --- | --- | --- | --- | --- | --- |
| 2. **Ngày giờ nhập viện:** [___][___]/[___][___]/[___][___] lúc [___][___]:[___][___]   *Ngày tháng năm Dùng định dạng 24 giờ* | | | | | | | | | | | |
| 3. **Nơi chuyển đến**   *Nếu đến từ bệnh viện khác, ngày nhập viện đầu tiên :* | | | | | | ⬜ Từ cộng đồng ⬜ Từ bệnh viện khác  [___][___]/[___][___]/[___][___] | | | | | |
| 4. **Ngày giờ nhập khoa Hồi sức tích cực ở bệnh viện hiện tại** : | | | | | | [___][___]/[___][___]/[___][___] lúc___][___]:[___][___]  *Ngày tháng năm Dùng định dạng 24 giờ* | | | | | |
| 5. **Ngày chuyển khỏi khoa ICU:**   **Tổng số ngày nằm ICU** | | | | | | [___][___]/[___][___]/[___][___](*nn/tt/nn*) [___][___][___] ngày | | | | | |
| 6. **Ngày xuất viện**: | | | | | | [___][___]/[___][___]/[___][___] ( *nn/tt/nn*) | | | | | |
| 7. **Kết cục**: ⬜ Tử vong ⬜ Xin về nhà/chuyển viện khác để tử vong ⬜ Chuyển viện ⬜ Xuất viện về nhà | | | | | | | | | | | |
| 8. **Các bệnh đồng mắc trước khi nhập viện (điền tất cả các hàng)** | | | | | | | | | | | |
| Cao Huyết Áp | | | | | ⬜ CÓ ⬜ KHÔNG ⬜ NA | |  | |  | | |
| Nhồi máu cơ tim | | | | | ⬜ CÓ ⬜ KHÔNG ⬜ NA | | Suy tim (độ I-III) | | ⬜ CÓ ⬜ KHÔNG ⬜ NA | | |
| Đau thắt ngực khi gắng sức nhẹ hoặc khi nghỉ ngơi | | | | | ⬜ CÓ ⬜ KHÔNG ⬜ NA | | Suy tim (độ IV) | | ⬜ CÓ ⬜ KHÔNG ⬜ NA | | |
| Bệnh mạch máu ngoại biên | | | | | ⬜ CÓ ⬜ KHÔNG ⬜ NA | | Bệnh mạch máu não | | ⬜ CÓ ⬜ KHÔNG ⬜ NA | | |
| Bệnh phổi mạn tính | | | | | ⬜ CÓ ⬜ KHÔNG ⬜ NA | | Suy hô hấp nặng | | ⬜ CÓ ⬜ KHÔNG ⬜ NA | | |
| Bệnh mô liên kết | | | | | ⬜ CÓ ⬜ KHÔNG ⬜ NA | | Bệnh viêm loét dạ dày | | ⬜ CÓ ⬜ KHÔNG ⬜ NA | | |
| Bệnh gan nhẹ | | | | | ⬜ CÓ ⬜ KHÔNG ⬜ NA | | Tiểu đường | | ⬜ CÓ ⬜ KHÔNG ⬜ NA | | |
| Liệt nửa người hoặc liệt hai chân | | ⬜ CÓ ⬜ KHÔNG ⬜ NA | | | | | Bệnh thận trung bình/ nặng | | ⬜ CÓ ⬜ KHÔNG ⬜ NA | | |
| Bệnh tiểu đường có tổn thương cơ quan đích | | | | ⬜ CÓ ⬜ KHÔNG ⬜ NA | | | Bệnh leukemia/khối u | | ⬜CÓ ⬜ KHÔNG ⬜ NA | | |
| Bệnh gan trung bình hoặc nặng/xơ gan | | | | ⬜ CÓ ⬜ KHÔNG ⬜ NA | | | Ung thư di căn | | ⬜CÓ ⬜ KHÔNG ⬜ NA | | |
| AIDS | | | ⬜ CÓ ⬜ KHÔNG ⬜ NA | | | | Sa sút trí tuệ | | ⬜CÓ ⬜ KHÔNG ⬜ NA | | |
| Khác 1 (ghi rõ) | | ____________________ | | | | | Khác 2 | | | | ________ |
| Phẫu thuật, mổ phiên trong vòng 30 ngày qua | | | | | | | ⬜CÓ ⬜ KHÔNG ⬜ NA | | | | |
| Phẫu thuật, mổ cấp cứu trong vòng 30 ngày qua | | | | | | | ⬜CÓ ⬜ KHÔNG ⬜ NA | | | | |
| 9. **Thời gian từ lúc có triệu chứng đầu tiên cho đến khi nhập viện** [__\|__] ngày | | | | | | | | | | | |
| 10. **Thời gian ủ bệnh** [__\|__] ngày N/A ⬜ | | | | | | | | **Thời gian toàn phát** [__\|__\|__] giờ | | | |
| 11. **Vết thương**: Nội tạng hay tiêm chích ⬜ | | | | | | | | Khác (hoặc không rõ) ⬜ | | | |
| 12. **Khó thở khi nhâp viện** | | | | | | | | Có ⬜ Không ⬜ | | | |
| 13. **Cân nặng_nếu có cân**: \|__\|__\|__] Kg | | | | | | | | **Ước lượng chiều cao** \|__\|__\|__] cm | | | |
| 14. **Chỉ số ABLETT** | ⬜ I *(không co giật)* ⬜ III *(co giật ảnh hưởng sự hô hấp)* ⬜ II *(co giật nhưng không ảnh hưởng sự hô hấp)* ⬜ IV *(RLTKTV)* | | | | | | | | | | |
| 15. **ASA** | Tình trạng sức khỏe tốt  Có bệnh hay tổn thương nhẹ  Có bệnh trung bình nặng  Có bệnh nặng nhưng không đe dọa tính mạng  Bệnh nặng đe dọa tính mạng tức thì | | | | | | | | | ⬜  ⬜  ⬜  ⬜  ⬜ | |
| 16. **Chỉ số SOFA/ APACHE/ TSS** | Giá trị xấu nhất trong 24g đầu ICU  Nhiệt độ cao nhất [__\|__\|.\|__]°C GCS [__\|**__]** RR [__\|__]lần/phút  FiO_2_ [__\|__\|% SpO_2_ [__\|__\|% PaO_2_ mmHg [__\|__\|__] pH [__\|__\|.\|__]  TC [__\|__\|__]10^3^/mm^3^ BC [__\|__\|__]10^9^/mm^3^ HCT [__\|__]%  Nhịp tim cao nhất [__\|__\|__] lần/phút thấp nhất [__\|__\|__] lần/phút  Huyết áp tâm thu cao nhất [__\|__\|__] mmHg  Huyết áp TB xấu nhất MAP [__\|__\|__]/[__\|__\|__]mmHg  Vận mạch Không⭘ Có⭘ Nếu có, tên thuốc …………………… liều …….. µg/kg/min  Bilirubin [__\|__\|__]mg/dL Na [__\|__\|__]mEq/L K [**__].**[__\|**__]**mEq/L  Creatinine [__\|__\|__] µmol/L Số lượng nước tiểu [__\|__\|__\|__]mL/24hr  Acute renal failure Có Không ⭘ Immunocompromised Có ⭘ Không ⭘ | | | | | | | | | | |

| 17. Ngày HTIG/SAT TB | [__\|__]/[__\|__]/[__\|__] | [__\|__]:[__\|__] |
| --- | --- | --- |
| SAT ở bệnh viện khác? | Có ⬜ Không ⬜ |  |
| 18. Ngày TKT | [__\|__]/[__\|__]/[__\|__] | [__\|__]:[__\|__] |
| 19. Biến cố bất lợi TB | Có ⬜ Không ⬜ |  |
| Nếu có ghi rõ: |  |  |
| 20. Biến cố bất lợi TKT | Có ⬜ Không ⬜ |  |
| Nếu có ghi rõ: |  |  |

| 1. **Bệnh nhân có từng mở khí quản?** ⬜Có ⬜ Không . ***Nếu có:***   Ngày giờ mở khí quản : [___][___]/[___][___]/[___][___] lúc [___][___]:[___][___]  *ngày tháng năm Dùng định dạng 24 giờ* | | |
| --- | --- | --- |
| *Chỉ định* |  | |
| Co thắt hầu họng thanh quản | ⬜ | |
| Ứ đàm | ⬜ | |
| Cần sử dụng thuốc dãn cơ và thở máy | ⬜ | |
| 2. **Ngày rút canuyn *:***   Lần 1: Ngày giờ: [___][___]/[___][___]/[___][___] lúc [___][___]:[___][___]  *ngày tháng năm Dùng định dạng 24 giờ*  **Có ngừng MKQ trên 1 đợt không?** ⬜ Có ⬜ Không *Thêm tờ bổ sung nếu ngừng hơn 1 lần* | | |
| 3. **Bắt đầu thở máy:** ⬜Có ⬜ Không [___][___]/[___][___]/[___][___] lúc ___][___]:[___][___]   *ngày tháng năm Dùng định dạng 24 giờ* | | |
| *Chỉ định* | |  |
| SaO_2_ <90% | | ⬜ |
| PaO_2_/Fi0_2_ <250 | | ⬜ |
| Co thắt dữ dội cần dùng thuốc dãn cơ | | ⬜ |
| 4. **Ngày ngừng thở máy*:* ?**   Lần 1: Ngày giờ: [___][___]/[___][___]/[___][___] lúc [___][___]:[___][___]  *ngày tháng năm Dùng định dạng 24 giờ*  Bệnh nhân có phải thở máy lại không ? ⬜ Có ⬜ Không ⬜ NA. Nếu có ***:***  Lần 2 ngày giờ:: [___][___]/[___][___]/[___][___] lúc [___][___]:[___][___]  *ngày tháng năm Dùng định dạng 24 giờ*  Ngày ngừng thở máy Lần 2?  Lần 2 ngày giờ:: [___][___]/[___][___]/[___][___]lúc [___][___]:[___][___]  *ngày tháng năm Dùng định dạng 24 giờ*  **Có ngừng thở máy trên 1 đợt không?** ⬜Có ⬜ Không *Thêm tờ bổ sung nếu ngừng hơn 1 lần* | | |

| **Viêm phổi liên quan đến thở máy (VAP) Ngày** (nn/tt/nn) **____/____/____**  BN được đặt NKQ/MKQ và thở máy trong vòng ít nhất 48 giờ và có 2 trong những tiêu chuẩn sau: | | |
| --- | --- | --- |
| + NĐ > 38°C hoặc < 36°C | ☐ Có ☐ Không | |
| + BC/máu < 4.0×10^9^/L hoặc ≥ 12×10^9^/L | ☐ Có ☐ Không | |
| + Đàm khí quản có mủ | ☐ Có ☐ Không | |
| + Thâm nhiễm mới hay nặng hơn trên phim Xquang phổi : | ☐ Có ☐ Không | |
| **VAP** | **☐ Có ☐ Không** | |
| **VAP xác định bằng vi sinh:** |  | |
| + Cấy đàm khí quản (ETA) có vi trùng > 10^5^ cfu/ml | **☐ Có ☐ Không** | |
| **Rối loạn thần kinh thực vật (RLTKTV) Ngày** (nn/tt/nn)**____/____/____**  BN có ít nhất 3 trong những tiêu chuẩn sau: | | |
| + M > 100 lần/ph | | ☐ Có ☐ Không |
| + Cao HA với HA tâm thu > 140 mmHg | | ☐ Có ☐ Không |
| + Tụt HA với HA tâm thu < 60 mmHg | | ☐ Có ☐ Không |
| + NĐ > 38°C | | ☐ Có ☐Không |
| + HA dao động (cao hay tụt) | | ☐ Có ☐ Không |
| **RLTKTV** | | **☐ Có ☐ Không** |
| **Loét giường** ☐ Có ☐ Không **Ngày** bệnh ____ | | |
| **Xuất huyết tiêu hóa** ☐ Có ☐ Không **Ngày** bệnh _**___** | | |
| **Khác** ☐ Có ☐ Không ................................................................................... | | |

| 1. **Ngày** (nn/tt/nn) | **____/____/____** | **____/____/____** | **____/____/____** | **____/____/____** | **____/____/____** | **____/____/____** | **____/____/____** |
| --- | --- | --- | --- | --- | --- | --- | --- |
| 2. **Nhiệt độ cao nhất** | [__\|__]**.**[__] ^o^C | [__\|__]**.**[__] ^o^C | [__\|__]**.**[__] ^o^C | [__\|__]**.**[__] ^o^C | [__\|__]**.**[__] ^o^C | [__\|__]**.**[__] ^o^C | [__\|__]**.**[__] ^o^C |
| 3. **HATT tối đa/thiểu mmHg** |  |  |  |  |  |  |  |
| 4. **Nhịp tim tối đa/thiểu l/p** |  |  |  |  |  |  |  |
| 5. **Midazolam TM** mg/d |  |  |  |  |  |  |  |
| 6. **Diazepam TM** mg/d |  |  |  |  |  |  |  |
| 7. **Diazepam uống** mg/d |  |  |  |  |  |  |  |
| 8. **Pipecuronium** mg/d |  |  |  |  |  |  |  |
| 9. **Fentanyl** mcg/d |  |  |  |  |  |  |  |
| 10. **Magnesium** g/hr |  |  |  |  |  |  |  |
| 11. **Thuốc vận mạch** |  |  |  |  |  |  |  |
| 12. **Thở máy +/-** |  |  |  |  |  |  |  |
| 13. **RLTKTV +/-** |  |  |  |  |  |  |  |
| 14. **Khang Sinh +/-** |  |  |  |  |  |  |  |
| 15. **Chỉ định** | O UV  O VPTM  O NT Tiểu  O NT Đ Máu  O Khác  ……………… | O UV  O VPTM  O NT Tiểu  O NT Đ Máu  O Khác  ……………… | O UV  O VPTM  O NT Tiểu  O NT Đ Máu  O Khác  ……………… | O UV  O VPTM  O NT Tiểu  O NT Đ Máu  O Khác  ……………… | O UV  O VPTM  O NT Tiểu  O NT Đ Máu  O Khác  ……………… | O UV  O VPTM  O NT Tiểu  O NT Đ Máu  O Khác  ……………… | O UV  O VPTM  O NT Tiểu  O NT Đ Máu  O Khác  ……………… |
| 16. **AE/SAE/USAE +/-** |  |  |  |  |  |  |  |
| 17. **Biến Chứng khác** |  |  |  |  |  |  |  |

| 1. **Ngày** nn/tt/nn) | **____/____/____** | **____/____/____** | **____/____/____** | **____/____/____** | **____/____/____** | **____/____/____** | **____/____/____** |
| --- | --- | --- | --- | --- | --- | --- | --- |
| 2. **Nhiệt độ cao nhất** | [__\|__]**.**[__] ^o^C | [__\|__]**.**[__] ^o^C | [__\|__]**.**[__] ^o^C | [__\|__]**.**[__] ^o^C | [__\|__]**.**[__] ^o^C | [__\|__]**.**[__] ^o^C | [__\|__]**.**[__] ^o^C |
| 3. **HATT tối đa/ thiểu mHg** |  |  |  |  |  |  |  |
| 4. **Nhịp Tim tối đa/thiểu l/p** |  |  |  |  |  |  |  |
| 5. **Midazolam TM** mg/d |  |  |  |  |  |  |  |
| 6. **Diazepam TM** mg/d |  |  |  |  |  |  |  |
| 7. **Diazepam uống** mg/d |  |  |  |  |  |  |  |
| 8. **Pipecuronium** mg/d |  |  |  |  |  |  |  |
| 9. **Fentanyl** mcg/d |  |  |  |  |  |  |  |
| 10. **Magnesium** g/hr |  |  |  |  |  |  |  |
| 11. **Thuốc vận mạch** |  |  |  |  |  |  |  |
| 12. **Thở máy +/-** |  |  |  |  |  |  |  |
| 13. **RLTKTV +/-** |  |  |  |  |  |  |  |
| 14. **Khang Sinh +/-** |  |  |  |  |  |  |  |
| 15. **Chỉ định** | O UV  O VPTM  O NT Tiểu  O NT Đ Máu  O Khác  ……………… | O UV  O VPTM  O NT Tiểu  O NT Đ Máu  O Khác  ……………… | O UV  O VPTM  O NT Tiểu  O NT Đ Máu  O Khác  ……………… | O UV  O VPTM  O NT Tiểu  O NT Đ Máu  O Khác  ……………… | O UV  O VPTM  O NT Tiểu  O NT Đ Máu  O Khác  ……………… | O UV  O VPTM  O NT Tiểu  O NT Đ Máu  O Khác  ……………… | O UV  O VPTM  O NT Tiểu  O NT Đ Máu  O Khác  ……………… |
| 16. **AE/SAE/USAE +/-** |  |  |  |  |  |  |  |
| 17. **Biến Chứng khác** |  |  |  |  |  |  |  |

1. **Bệnh nhân đã hoàn thành nghiên cứu?**

⬜ Có

⬜ Không, không đũ tiêu chuẩn nhận vào, ngày [___][___]/[___][___]/[___][___](*nn/tt/nn*)

⬜ Không, bệnh nhân rút chấp thuận tham gia ngày [___][___]/[___][___]/[___][___](*nn/tt/nn*)

⬜ Không, do bác sĩ kết thúc nghiên cứu ngày [___][___]/[___][___]/[___][___](*nn/tt/nn*)

⬜ Không, bệnh nhân tử vong/ xin về

⬜ Không, không tuân thủ diêụ trị

⬜ Không, di ứng thuốc

⬜ Không, không thể chọc dọ dịch não thủy

*Nếu không, ngày rút khỏi nghiên cứu:*

2. **Viện phí trong thời gian điều trị tại khoa HSTC :**

[___] [___][___][___] [___][___][___] [___][___][___] Đồng

3. **Tổng viện phí:**

[___] [___][___][___] [___][___][___] [___][___][___] Đồng

| 1. **Ngày theo dõi thứ 240**: Ngày:[___][___]/[___][___]/[___][___] (nn/tt/nn) 2. **Có liên lạc được**: ⬜ Có ⬜Không   Lý do không liên lạc được: ______________________________________________-   3. **Địa điểm bệnh nhân ở hiện tại:**   ⬜ Đã tử vong Ngày tử vong :[___][___]/[___][___]/[___][___] (nn/tt/nn)  Lý do tử vong: ……………………………….  Nếu sống: ⬜ Tại nhà hoặc nhà người thân ⬜ Tại bệnh viện này ⬜ Tại bệnh viện khác | |
| --- | --- |
| 4. **Bệnh nhận trở lại công việc**   ⬜ Có    ⬜ Không | ⬜ Có, như trước khi bị bệnh  ⬜ Có, nửa thời gian trước đây/ giảm giờ làm việc |
| 5. **Có biến chứng thần kinh?**   ⬜ Có    ⬜ Không | ⬜ yếu cơ  ⬜ cứng cơ  ⬜ rối loạn cảm giác  ⬜ Khác ……………………………………………. |
| 6. **Rankin**   ⬜ 0: Không triệu chứng  ⬜ 1: vài triệu chứng nhẹ không ảnh hưởng đến cuộc sống.  ⬜ 2: có các triệu chứng làm hạn chế cuộc sống nhưng bệnh nhân vẫn tự chăm sóc bản thân được.  ⬜ 3: các triệu chứng làm hạn chế cuộc sống ngăn trở sống độc lập.  ⬜ 4: các triệu chứng ngăn trở sống độc lập một cách rõ rệt, dù chưa đòi hỏi phải được chú ý, săn sóc đặc biệt thường xuyên.  ⬜ 5: phụ thuộc hoàn toàn vào người khác, cần dược săn sóc, giúp đở ngày đêm.  ⬜ 6: chết | |
| 7. **Vấn đề khác** | |
