# Supplementary material for: Intrathecal Immunoglobulin for treatment of adult patients with tetanus: A randomized controlled 2x2 factorial trial
Source: Wellcome Open Res. 2018 Nov 5;3:58. Originally published 2018 May 16. [Version 2] doi: 10.12688/wellcomeopenres.14587.2 (PMC6372971; doi:10.12688/wellcomeopenres.14587.2)
Supplement: Peer review history

# Peer review: Referee response for version 2

**DOI:** 10.21956/wellcomeopenres.16235.r34739

**Published:** 2019-02-08

**Authors:** Philip Bejon

**Affiliations:** 1 Centre for Geographic Medicine Research-Coast, KEMRI-Wellcome Trust Research Programme, Kilifi, Kenya

**Overview**

 The protocol covers two randomizations for potential tetanus treatments; a) intrathecal anti-tetanus immunoglobulin vs sham procedure and b) human vs equine anti-tetanus immunoglobulin.

 The primary outcome is the requirement for ventilation.

 The findings of this study could be applicable immediately to clinical protocols and therefore the study promises impact. The design seems appropriate and I have only a few comments and suggestions.

**Background**

 The background appropriately points out that tetanus is vaccine preventable. It could be further discussed that the problem with vaccination appears not to be coverage in childhood, but boosting to maintain immunity in later life, and preventing neonatal tetanus requires maternal vaccination. Otherwise the vaccination coverage seems at odds with the statement that a substantial proportion of the population in SE Asia remains susceptible. The GBD data suggest that rates of tetanus have fallen substantially in many areas, and our own local data ^1^ is consistent with that trend. 

 Should the argument be addressed whether despite these encouraging trends that some adult tetanus will remain and therefore optimal treatment is required? Some will think that time and effort would be better spent on vaccine coverage rather than in ICUs.

 Could the overall number of patients randomized to date across the trials be stated so this can be compared with the power added by the trial here?

 The introduction states “only one blinded trial” but the protocol is partially blinded. Were other trials partially blinded?

**Trial**

 In addition to the secondary endpoints listed, should the investigators consider whether they can detect any bias in terms of the treatment decision? 

 For instance, either the criteria currently used for decisions, or the arterial CO _2_, which could theoretically indicate a higher threshold for ventilating patients in the intervention group if there was a bias against ventilating because of unwarranted confidence among clinicians due to insufficient blinding? 

**Informed consent**

 I would imagine that most patients will not have the capacity to give consent at the point of randomization. Have the authors had difficulty taking consent from relatives in what must be a very charged situation?

 CO _2_ monitoring doesn’t seem to be mentioned, but should be routinely available and might be valuable data?

 What is the definition of “independent” for the doctor/nurse for purpose of blinding. Are they from a different institution? Or a paid locum?

**Statistics**

 The number of patients sounds very high. From what referral population do these come? 

 The sample size calculation doesn’t consider the possibility of an interaction. Likely not much can be seen if there is an interaction as stated in the text, but would be useful to at least know the power under those circumstances since it is a planned analysis.

 What about a planned sensitivity analysis where ventilation decisions taken outside these criteria are excluded, or where different case definitions are applied to the criteria at the point of ventilating?

**Discussion**

 The study is single centre. Could the authors comment on likely generalizability? Are there any other centres that could conceivably recruit similar numbers?

I have read this submission. I believe that I have an appropriate level of expertise to confirm that it is of an acceptable scientific standard.

## References

[1] IbindaFBauniEKariukiSMFeganGLewaJMwikambaMBogaMOdhiamboRMwagandiKSealeACBerkleyJADorfmanJRNewtonCR: Incidence and risk factors for neonatal tetanus in admissions to Kilifi County Hospital, Kenya. *PLoS One*.2015;10(4) : 10.1371/journal.pone.0122606 e0122606 10.1371/journal.pone.0122606 25849440PMC4388671

# Peer review: Referee response for version 2

**DOI:** 10.21956/wellcomeopenres.16235.r34187

**Published:** 2018-11-05

**Authors:** Joanne M. Langley

**Affiliations:** 1 Departments of Pediatrics, and Community Health & Epidemiology, Canadian Center for Vaccinology, Dalhousie University, Halifax, NS, Canada

I have read this submission. I believe that I have an appropriate level of expertise to confirm that it is of an acceptable scientific standard.

# Peer review: Referee response for version 1

**DOI:** 10.21956/wellcomeopenres.15882.r33666

**Published:** 2018-10-09

**Authors:** Joanne M. Langley

**Affiliations:** 1 Departments of Pediatrics, and Community Health & Epidemiology, Canadian Center for Vaccinology, Dalhousie University, Halifax, NS, Canada

This paper describes the protocol for a randomized controlled study of intramuscular (IM) immunoglobulin Ig, either equine or human with or without intrathecal human Ig. The primary outcome measure is the proportion of patients in each group that requires mechanical ventilation.

 Questions and comments:

1. The introduction refers to “severe tetanus” patients who require admission to an ICU for mechanical ventilation and paralysis. What is “severe tetanus”? Similarly patients with “generalized tetanus” are eligible. Descriptions of these terms are needed so that future readers can determine if the results apply to their patient population.
2. An inclusion criterion for the study is admission to the ICU; if so then wouldn’t all participants require mechanical ventilation, which is the primary outcome measure?
3. How is “autonomic dysfunction syndrome” defined.
4. Participant allocation in Figure 1 does not align with the text. (There are two boxes with equine antitoxin + intrathecal human antitoxin)
5. The flow chart would further align with the text if the first decision point were human v equine antitoxin (see wording in 2, para 1), and then 4 study groups arise based on allocation of intrathecal treatment or not. Alternatively this text could state that participants are randomized 1:1:1:1 to one of four study groups
6. It is not clear if study physicians are in any way involved in patient care or not, i.e. are distinct from clinical care staff.
7. 2.1 States the criteria for mechanical ventilation. Since the decision to intervene is up to the clinical staff, suggest rewording this to “clinical staff will provide routine care to patients, including decisions as to whether intubate and ventilate”. Are other forms of respiratory support used in the unit (e.g. CPAP)  and if so will this be recorded.
8. Typo 3.4 “versions” not “version”
9. Typo 3.4 “assent” not “ascent”
10. 3.4 Informed consent. “Patients will be withdrawn from the study if they do not give …consent..” How would they be included in the study if they have not given consent?
11. A secondary outcome is occurrence of ventilator associated pneumonia. It is not possible for Patients who are not ventilated (the primary outcome) to have this outcome.
12. Will the doses of benzodiazepams be adjusted for patient weight and co-morbidities for which these drugs could be prescribed?
13. Patients who receive IM antitoxin before arrival at the study hospital are eligible for enrolment. These patients will be unblinded.
14. 3.7 indicates ward nurses are responsible for administration of study product. Will they be unblinded or are individual syringes masked?
15. Regarding the sham procedure, will a second staff member be present to secure the patient during the LP? Has thought been given to how the blind will be maintained? Will the same amount of time be taken to do a sham procedure as a true procedure?
16. Re: daily management. What are the defined protocols for ventilation. These could change the risk of VAP if there is variability in implementation.
17. Why are antibiotics routinely used (this is implied)
18. 3.10. Typo “measured” not “measure”
19. 3.10 Typo “spasm” not “spam”
20. It is not clear which AEs are solicited v unsolicited, how long these will be collected, and what the gradation for these outcomes will be.
21. 4.4. Typo “SAEs” not “SEAs”
22. Why is the DSMB viewing unblinded data? They could review it by study groups 1-4.
23. Are there pausing rules?
24. The cost-effectiveness analysis appears first in the statistical section. It should appear in the endpoints.
25. How is the follow up to day 240 being done. Is it expected that all patients will still be in hospital?
26. The risk of equine antitoxin is described variously as theoretical and infrequent (<1%). Please clarify.

I have read this submission. I believe that I have an appropriate level of expertise to confirm that it is of an acceptable scientific standard, however I have significant reservations, as outlined above.

# Response

**Published:** 2018-10-23

**Authors:** C Louise Thwaites

**Affiliations:** Oxford University Clinical Research Unit, Vietnam

Thank you for your very careful and thoughtful review of our protocol. We have made some modifications in line with the comments, but responses to specific points are addressed below:

1. Severe tetanus has been clarified to indicate the common understanding of this term – ie frequent spasms or local laryngeal spasms, similarly generalized tetanus – one of the recognized 2 forms of tetanus (generalized, localized)

2.  Requirement of mechanical ventilation is not a necessary condition for admission to ICU. Current policy in our hospital requires that all patients with tetanus are initially admitted there as the disease evolves over several days and sudden deterioration can be quickly acted upon. Only approximately 50% of those admitted to the ICU require mechanical ventilation (see sample size currently).  

3. In the protocol this is defined as ‘clinical incidence of autonomic nervous system disturbance’ meaning that it depends on treating clinicians judgement. However in our CRF we are collecting detailed information on criteria used to make this judgement and these are the same criteria used in our previous clinical trial [Thwaites et al, Lancet 2006].

4/5. This has been corrected

6.The study physicians are not involved in patient care in any way. They do not work in the ICU department. This has been further clarified in the discussion section

7. Thank you. This protocol is currently approved by 3 different ethical committees and has recruited over 150 patients and therefore cannot be changed at this point. With respect to CPAP, this is never used for tetanus patients in our hospital (Hospital for Tropical Disease guidelines) Spasm is the main cause of ventilator failure in these patients and therefore CPAP is not used.   

8. This has been corrected.

9. This has been corrected

10.Consent may previously have been given by relatives whilst the patient was unable to give consent themselves.

11.Correct – statistical reporting will account for this. Nevertheless previous studies have shown this is one of the most important factors related to increased cost and length of stay of patients who are mechanically ventilated and therefore should be collected. 

12. These drugs are given and titrated against spasm control and are not given on a mg/kg basis, therefore correcting for these factors will not be done. We will make this clear in the final study write-up

13.They will be unblinded for the intramuscular allocation only but the primary comparison of the intrathecal allocation will still be blinded. We have gone to great lengths to ensure that blinding is maintained for this group.

14.They are fully blinded as described above – working in a different department and fully trained in all the study procedures. They remove all study product from the ward after administration so ICU staff do not know which treatment has been given.

15. Yes – 2 nurses and one doctor (all independent from a different department) administer the LP or sham behind screens. They follow the same protocol and take the same length of time.

16. There is a standard ventilation procedure that is followed within the ICU at the Hospital for Tropical Diseases and patients with tetanus are treated according to standard guidelines, thus it is unlikely any differences will occur. To my knowledge there is no published evidence of ventilation protocols affecting the likelihood of VAP. All weaning follows a standard process of reducing sedatives and muscle relaxants followed by spontaneous breathing trial.

17. Metronidazole is given to all patients for treatment of *Clostridium tetani* infection. Antibiotics are otherwise only given for specific indications. In section 3.10 new antibiotic prescriptions are collected in addition to nosocomial infection data. Previous clinical studies of nosocomial infection we have used have collected both data as both are valuable. ICU infections are notoriously difficult to diagnoses (eg VAP) and sometimes antibiotics are given without a clear indication but this is nevertheless an important endpoint (and perhaps even more important).

18. This has been corrected

19. This has been corrected

20. Patients are under active surveillance for AEs whilst in hospital. Telephone follow up of known AEs then continues after discharge. For completeness all AEs and medical interventions are recorded and reviewed by an AE committee. SAEs are reported as described.

21. This has been corrected

22. You are correct the DSMB are viewing as groups 1-4 but also if necessary can have access to more detailed information as they require.

23.The DSMB follow a pre-defined charter. They have not, as yet, published to us any pre-demfined pausing rules.

24. This is correct. The cost data is collected in all patients.

25. This is being done by telephone – it is expected that most patients will be at home. Most patients live at very great distances from the hospital but previous experience has shown >90% follow-up rates after 6 months.

26. There is to my knowledge no published data giving incidence of equine antitoxin side effects using current preparations. We here have based our infrequent on an internal assessment within our ICU as stated.
